# Conditions for Eltonian Pyramids in Lotka-Volterra Food Chains

**DOI:** 10.1038/s41598-017-11204-1

**Published:** 2017-09-07

**Authors:** Tomas Jonsson

**Affiliations:** 1 Ecological Modeling Group, School of Bioscience, University of Skövde, Box 408, SE-541 28, Skövde, Sweden; 2 Department of Ecology, Swedish University of Agricultural Sciences, Box 7044, SE-750 07, Uppsala, Sweden

## Abstract

In ecological communities consumers (excluding parasites and parasitoids) are in general larger and less numerous than their resource. This results in a well-known observation known as ‘Eltonian pyramids’ or the ‘pyramid of numbers’, and metabolic arguments suggest that this pattern is independent of the number of trophic levels in a system. At the same time, Lotka-Volterra (LV) consumer-resource models are a frequently used tool to study many questions in community ecology, but their capacity to produce Eltonian pyramids has not been formally analysed. Here, I address this knowledge gap by investigating if and when LV food chain models give rise to Eltonian pyramids. I show that Eltonian pyramids are difficult to reproduce without density-dependent mortality in the consumers, unless biologically plausible relationships between mortality rate and interaction strength are taken into account.

## Introduction

A common observation in many ecosystems is that consumers are both larger and less abundant than their resources^1, 2^ (if parasites and parasitoids are ignored^3^). The resulting graph of numerical abundances versus trophic position produces the well-known ‘pyramid of numbers’^4, 5^, also termed ‘Eltonian pyramids’ in honour of C. Elton who almost 90 years ago^6^ drew attention to the observation that “... animals at the base of a food chain are relatively abundant, while those at the end are relatively few in numbers...”. The concept of Eltonian pyramids is not restricted to a pyramidal pattern in the distribution of numerical abundance however, but can also imply a pyramidal pattern in the distribution of biomass abundance (i.e. a pyramid of biomass).

Exceptions to a pyramidal view of life certainly exist, such as the number of herbivorous insects exceeding the number of trees in terrestrial forest ecosystems, a barrel-shaped distribution of biomass in a freshwater food web^1^ or even inverted biomass pyramids in some marine systems^7^. However, it can easily be shown, using metabolic arguments, that the shape of the stack of trophic level abundances, if assuming mainly bottom-up control, depends on (*i*) the ratio, ${\rho }_{T}={\overline{W}}_{T+1}/{\overline{W}}_{T}$, in average body size ($\overline{W}$) between organisms on adjacent trophic levels (*T* and *T* + 1) and (*ii*) the efficiency of energy transfer (*γ*_*T*_) between trophic levels (see Eqs S1.6 and S1.7, Note S1 in Supplementary Information). Ratios greater than unity (*ρ* > 1) and ecological (in)efficiencies (i.e. *γ* < 1) suffices to produce pyramids of numbers, while *γ* needs to be smaller than *ρ* ^*β*−1^ (where *β* is the metabolic exponent) to produce pyramids of biomass (Note S1). Acknowledging that consumers can reduce the abundance of their resources and thus affect production at the level below (i.e. allowing some degree of top-down control) does not eliminate the key roles of the consumer-resource body size ratio and ecological efficiencies for the shape of Eltonian pyramids. Although the expressions describing the metabolic constraints are slightly more complicated (Eqs S1.10 and S1.11), the main conclusion is that non-Eltonian pyramids of numerical abundance should be very rare, while non-Eltonian pyramids of biomass abundance can be expected to be more frequent under certain conditions, i.e. for consumers that are several orders of magnitude larger than their resource and/or significantly reduces the abundance of their resource (Note S1). Thus, a general picture from both metabolic considerations and empirical data across both terrestrial and aquatic biomes^8, 9^ is that trophic pyramids of numerical abundance are the norm and below I focus on these.

If Eltonian pyramids of numbers are the rule rather than the exception in ecological communities, mathematical representations of the processes that produce them (i.e., food chain or food web models) should be able to reproduce this pattern in a realistic way. Teramoto^10^ pioneered the study of this problem by analysing conditions for pyramidality in linear food chains with Lotka-Volterra (LV) dynamics. He showed that when such a system has the highest possible number of trophic levels (under given conditions of productivity and efficiency of energy transfer), the trophic levels always exhibit a pyramid-type structure, and drew attention to the importance of density-dependent competitive interactions for pyramidality. Here, I build and expand on these results by further investigating the capacity of LV food chain models to produce feasible food chains, where the pattern of trophic level equilibrium abundances is pyramidal, and highlighting conditions that impedes or facilitates this.

To understand the implications of these results, it is important to recognize that the metabolic based conditions for pyramidality above (based on Note S1) are entirely independent of the *number* of trophic levels in a community, as well as *what* trophic levels are considered. This means that gaining or losing a trophic level to/from a community, although most likely causing a trophic cascade that alters abundances of species and trophic levels, is not predicted to disrupt the pyramidal pattern of abundance and by extension, a community that is pyramidal when having *n* number of trophic levels, should be pyramidal for any other number of trophic levels (provided that values of *ρ* and *γ* do not violate the constraints above). This suggests that food chain models should have this property too, i.e. if a model food chain of length *n* is pyramidal, it should be able to gain and/or loose, a trophic level and still be pyramidal. Based on this, I here analyse the parameter conditions that allow and prohibit LV food chains to have this property, by specifically analysing the role of density dependent consumer mortality and constant vs. changing trophic interaction strengths across trophic levels, for allowing LV food chains of different lengths to be pyramidal, so that a food chain can both gain and loose a trophic level and still be pyramidal.

### Lotka-Volterra food chains

Ever since first formulated, independently by Lotka^11^ and Volterra^12^, the differential equations describing the coupled population dynamics of consumers and resources have been instrumental in developing and analysing hypotheses in many areas of community ecology. Initially, they were applied to one-consumer – one-resource systems, assuming a linear functional response of the consumer, but were later modified with saturating functional responses^13^, expanded to food chains to study trophic cascades^14^ and applied to multispecies systems to study dynamical and stability properties of food webs^15–19^. However, assuming that linear interspecific interactions is an approximation, in the vicinity of an equilibrium, of something more complex and non-linear, the original LV formulation with a linear consumer functional response still remains a standard approach when analysing consumer-resource systems ranging from simple (few species) biological control^20–22^ and consumer-resource^23–25^ modules, to multispecies food webs^26–28^ as well as addressing questions of evolutionary stability of ecological hierarchies^29^. Thus, since LV consumer-resource models with linear consumer functional response continue to be a useful modelling approach to address questions in community ecology it is relevant to study if and under what circumstances its ‘pyramidal predictions’ are realistic. Here, the pattern of trophic level abundances at equilibrium is analysed in linear LV food chains (without omnivory) with linear consumer functional responses (see Supplementary Information and discussion, however, for complementary results based on a saturating functional response):

1 $$\{\begin{array}{rcl}\frac{d{N}_{1}}{dt} & = & {N}_{1}({b}_{1}-{a}_{11}{N}_{1}-{a}_{12}{N}_{2})\\ \frac{d{N}_{2}}{dt} & = & {N}_{2}(-{b}_{2}-{a}_{22}{N}_{2}+{a}_{21}{N}_{1}-{a}_{23}{N}_{3})\\  &  & \vdots \\ \frac{d{N}_{n}}{dt} & = & {N}_{n}(-{b}_{n}-{a}_{nn}{N}_{n}+{a}_{n,n-1}{N}_{n-1})\end{array}$$

*N*_*i*_ (*i* = 1:*n*) is the abundance of trophic level (or species) *i*, *b*_*i*_ the per capita birth (*i* = 1) or death (*i* ≥ 2) rates and *a*_*ij*_ (*i* = 1:*n* − 1, *j* = *i* + 1, and *i* = 2:*n*, *j* = *i* − 1) the trophic interaction strengths. The consumer interaction strength is *a*_*i,i*+1_ (*i* = 1, … *n*−1) and the resource interaction strength is *a*_*i*+1,*i*_ (*i* = 1, … *n*−1). Direct density dependence enters via *a*_*ii*_ (*i* = 1:*n*). Trophic level (species) 1 is the producer while trophic levels 2 to *n* consist of consumers. Although phrased here as describing the dynamics in the *numerical* abundance of trophic levels (species), little in the analyses that follow precludes this formulation to be interpreted instead as describing the dynamics in the *biomass* abundance of trophic levels (species). However, since, as described above, non-pyramidal distributions of biomass can be expected to occur, while they should not for numerical abundance. I focus in what follows on numerical abundance. I furthermore stress that I am here not considering the abundance dynamics of individual populations or species, only the predicted abundances of trophic levels at equilibrium. The rationale for this, in addition to having a simpler system to analyse, is that any food web structure can, from an energy flow perspective, be collapsed to a linear trophic chain, by ‘unfolding its energy-flow network’^10^ (i.e. partitioning the biomass of each species/node to different trophic levels according to the path lengths of its origin). Thus, here I study the expected distribution of trophic level equilibrium abundances in LV food chains with different number of trophic levels.

In the original two-species formulation of Eq. (1) by Volterra^12^ neither resource, nor consumer, showed direct density dependence. Later modifications of the model (as seen in Eq. (1)) include resource logistic growth (via *a*_11_) and the potential for density-dependent consumer mortality (DDCM) via *a*_*ii*_. However, many investigations using the LV approach^20–22, 30, 31^ as well as overviews of trophic interactions in textbooks and elsewhere^4, 5, 32–36^ still follow Pimm and Lawton^37, 38^ in assigning density-dependent mortality to the producer level only (see however^19, 39–45^). Furthermore, it has become increasingly obvious that real ecological communities are characterized by a specific, non-random patterning of the magnitudes of ecological rates and species interactions^15, 42, 46^, with, among other things, mortality rates (*b*_*i*_) and trophic interaction strengths (*a*_*ij*_) suggested to be allometrically related to body size^26, 47^. All of this raises the question of if and how DDCM and body size effects on mortality rates and trophic interaction strengths affects the capacity of LV food chains to produce a ‘consistent pyramidal pattern of abundances’ (i.e. across food chains with different number of trophic levels). To address this I analyse the role of (*i*) DDCM, (*ii*) mortality rates and (*iii*) trophic interaction strengths in LV food chain models, for a pyramidal pattern of abundance, across food chains with different number of trophic levels, to emerge. It is argued that a community model should be able to generate such a pattern in order to be realistic and it is shown that all of the three mechanisms analysed (presence of DDCM, changing mortality rates and changing interaction strengths with increasing trophic position) increases the probability for a pyramidal pattern of abundance by increasing the ‘pyramidal parameter space’ for LV food chains of a particular length, as well as overlap in the pyramidal parameter spaces of food chains of different lengths.

## Results

**Figure 1:**
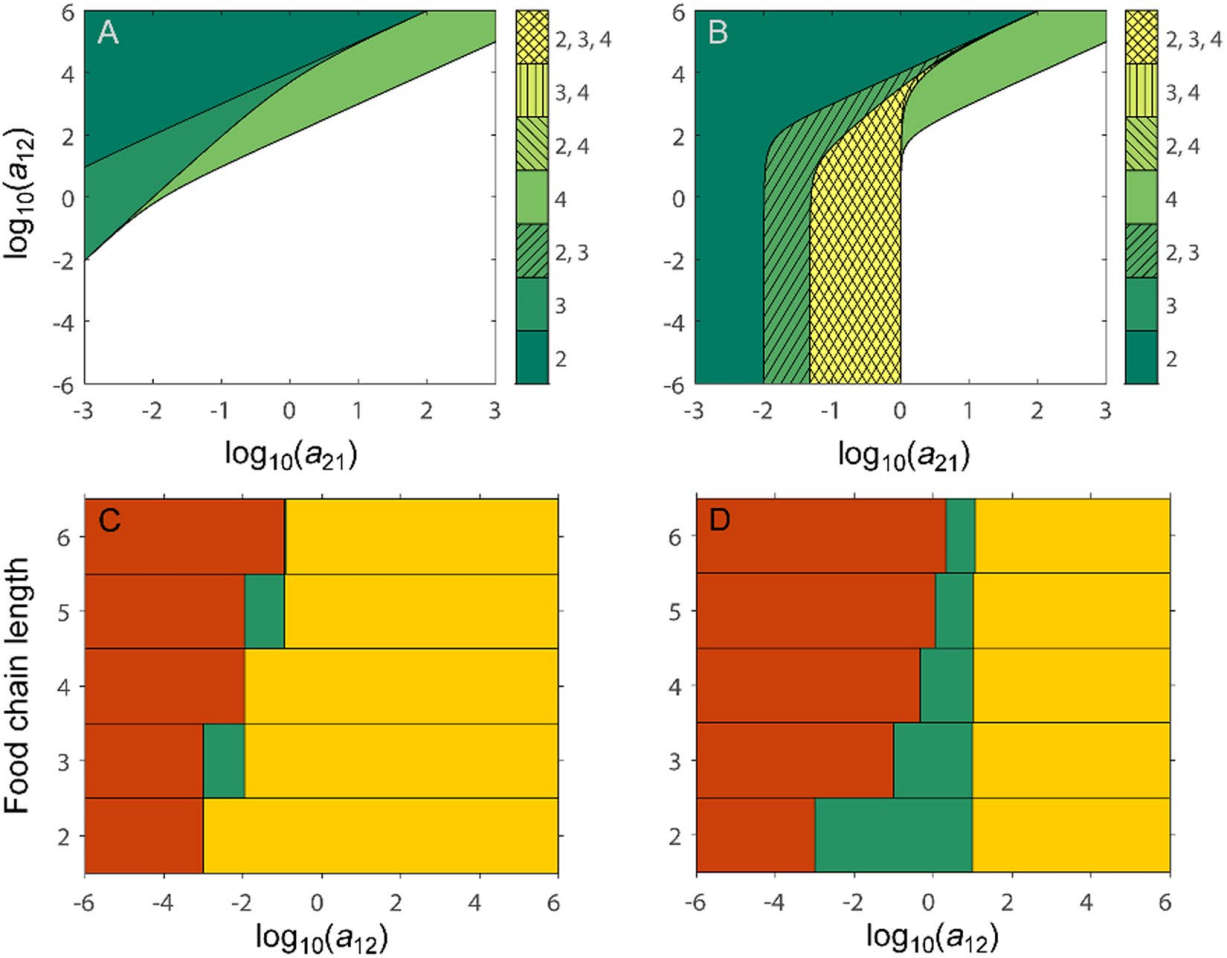
The presence of Eltonian pyramids in Lotka-Volterra (LV) food chains with different number of trophic levels, under the assumption of constant consumer mortalities and interaction strengths with trophic position. (**A**,**B**) Subplots show, using different colours and hatching, the regions in parameter space (i.e. combinations of prey, *a*_21_, and predator, *a*_12_, interaction strengths) where LV food chains of different lengths are pyramidal. For some combinations of *a*_21_ and *a*_12_ only one food chain length will produce Eltonian pyramids (=unhatched sectors, e.g. two trophic level food chains within dark green sector), while for other combinations of *a*_21_ and *a*_12_ more than one food chain length will produce Eltonian pyramids (=hatched sectors, e.g. both two and four, but not three trophic level food chains within light green, diagonally hatched sector). See colourbar for which lengths of a food chain that are pyramidal within each sector. (**C**,**D**) Subplots show, using horizontal bars of different colours, the range of values of consumer (*a*_12_) interaction strengths for which LV food chains with two to six trophic levels are (*i*) non-feasible (red region), (*ii*) feasible and pyramidal (green region), and (*iii*) feasible but non-pyramidal (yellow region), respectively, when resource and consumer interaction strengths are assumed to be related via the ecological efficiency (i.e. *a*_*i*+1*,i*_ = *γ* × *a*_*i,i*+1_). When green bars for two or more food chain lengths overlap vertically this means that food chains with these number of trophic levels all will be pyramidal for the range of values of *a*_12_ for which there is an overlap, while if there is no vertical overlap in green bars LV food chains of different lengths cannot be pyramidal simultaneously for any value of *a*_12_ used. Density-dependent consumer mortality is absent from food chains in (**A**,**C**) but included in food chains in (**B,D**). (See Figs S1, S2 for individual plots and a summary plot of the pyramidal parameter space for LV food chains with up to six trophic levels). Parameter settings: (**A**) *a*_11_ = 1, *a*_*ii*_ = 0 (*i* ≥ 2), *b*_1_ = 1, *b*_*i*_ = 0.0001 (*i* ≥ 2), *a*_*i,i+*1_ = *a*_12_ and *a*_*i*+1,*i*_ = *a*_21_, (**B**) Same as (**A**) except for *a*_*ii*_ = 1 (*i* ≥ 2), (**C**) Same as (**A**) except for *a*_*i*+1*,i*_ = *γ* × *a*_*i,i+*1_ with *γ* = 0.1, (**D**) Same as (**B**) except for *a*_*i*+1*,i*_ = *γ* × *a*_*i,i*+1_ with *γ* = 0.1.

**Figure 2:**
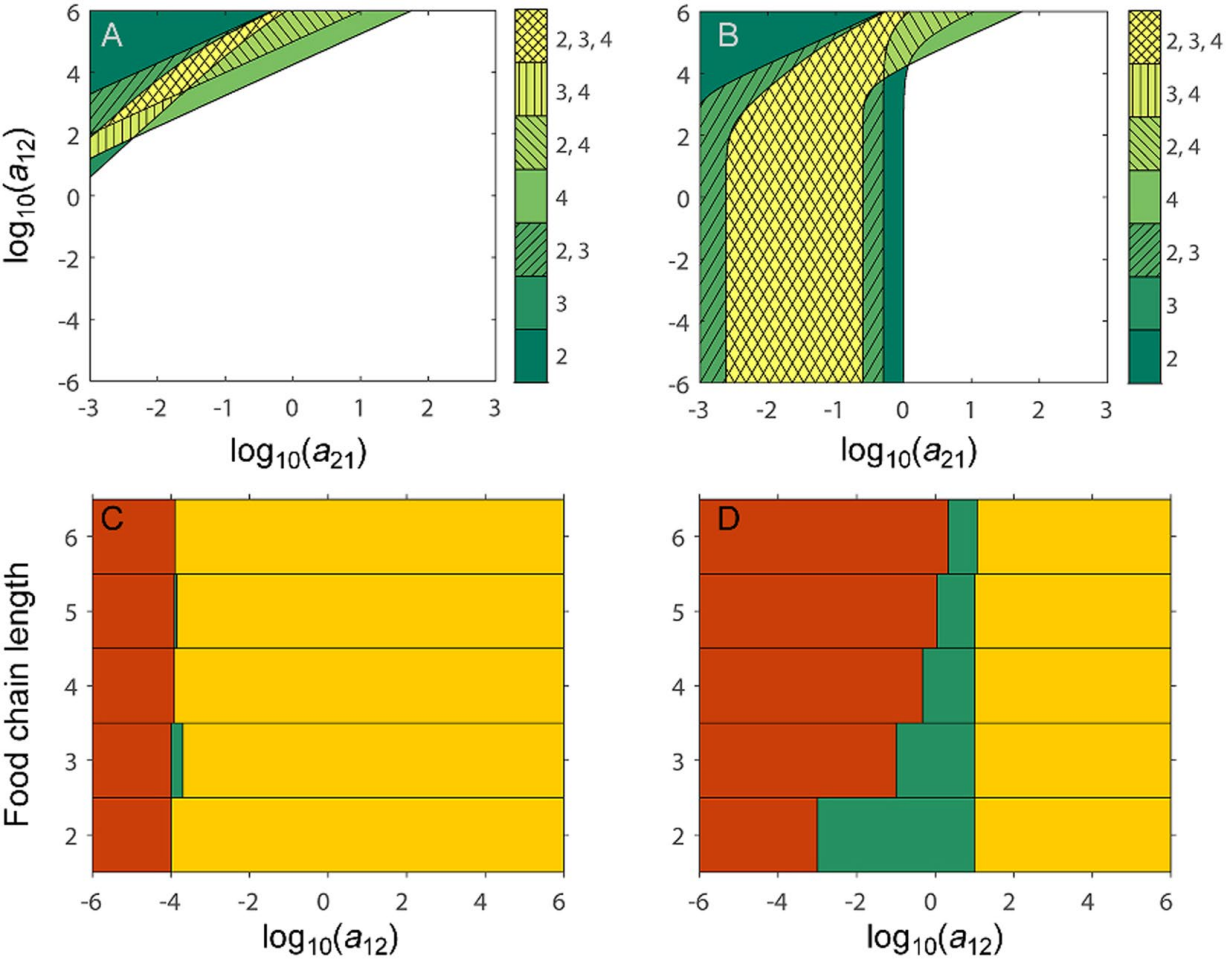
The presence of Eltonian pyramids in Lotka-Volterra (LV) food chains with different number of trophic levels, under the assumption of changing consumer mortalities and interaction strengths with trophic position. (**A**,**B**) Subplots show, using different colours and hatching, the regions in parameter space (i.e. combinations of prey, *a*_21_, and predator, *a*_12_, interaction strengths) where LV food chains of different lengths are pyramidal. For some combinations of *a*_21_ and *a*_12_ only one food chain length will produce Eltonian pyramids (=unhatched sectors, e.g. two trophic level food chains within dark green sector), while for other combinations of *a*_21_ and *a*_12_ more than one food chain length will produce Eltonian pyramids (=unhatched sectors, e.g. both two and four, but not three trophic level food chains within light green, diagonally hatched sector). See colourbar for which lengths of a food chain that are pyramidal within each sector. (**C**,**D**) Subplots show, using horizontal bars of different colours, the range of values of consumer (*a*_12_) interaction strengths for which LV food chains with two to six trophic levels are (*i*) non-feasible (red region), (*ii*) feasible and pyramidal (green region), and (*iii*) feasible but non-pyramidal (yellow region), respectively, when resource and consumer interaction strengths are assumed to be related via the ecological efficiency (i.e. *a*_*i*+1*,i*_ = *γ* × *a*_*i,i*+1_). When green bars for two or more food chain lengths overlap vertically this means that food chains with these number of trophic levels all will be pyramidal for the range of values of *a*_12_ for which there is an overlap, while if there is no vertical overlap in green bars LV food chains of different lengths cannot be pyramidal simultaneously for any value of *a*_12_ used. Density-dependent consumer mortality is absent from food chains in (**A**,**C**) but included in food chains in (**B**,**D**). (See Figs S5 and S6 for individual plots of the pyramidal parameter space for LV food chains with two to four trophic levels and a summary plot of food chains with up to six trophic levels). Parameter settings: (**A**) *a*_11_ = 1, *a*_*ii*_ = 0 (*i* ≥ 2), *b*_1_ = 1, ${b}_{i}={k}_{b}^{i-2}{b}_{2}(i\ge 2)$, ${a}_{i+1,i}={k}_{res}^{i-1}{a}_{2,1}$ and ${a}_{i,i+1}={k}_{cons}^{i-1}{a}_{1,2}$, where *k*_*b*_ = 0.1, *k*_*res*_ = 2 and *k*_*cons*_ = 0.5. (**B**) Same as (**A**) except for *a*_*ii*_ = 1 (*i* ≥ 2), (**C**) Same as (**A**) except for *a*_*i+*1*,i*_ = *γ* × *a*_*i,i*+1_ with *γ* = 0.1, (**D**) Same as (**B**) except for *a*_*i*+1*,i*_ = *γ* × *a*_*i,i*+1_ with *γ* = 0.1.

Below, simulation results of parameter combinations that allow for pyramidality in LV food chains, under different assumptions, are first presented graphically (Figs 1, 2). Next, analytical results (Notes S2–S4) are briefly summarized that generalize and support these simulation results. Finally, these results have been complemented by simulations where model parameters (mortality rates and interaction strengths) have been drawn at random from specific intervals, instead of being assigned certain values. The latter results are briefly summarized below (but can be found in full in Note S5).

### Constant mortality rates and interaction strengths

As a simple introductory case, assume that across trophic levels all of the consumer LV interaction strengths (the *a*_*ij*_’s) are of the same fixed value as well as all of the resource LV interaction strengths (the *a*_*ji*_’s), but without any relationship between *a*_*ij*_ and *a*_*ji*_. The combinations of parameters *a*_12_ and *a*_21_ that, under these assumptions (see Methods), give rise to Eltonian pyramids in LV food chains of length two to four, without and with DDCM respectively, are shown in Fig. 1A,B. Here, each filled sector (hatched and/or coloured) encompass a region in parameter space (combinations of *a*_12_ and *a*_21_) where some food chains will be pyramidal, with different colours and hatching separating parameter combinations that will give rise to pyramidality in food chains with two, three and/or four trophic levels. (See Figs S1 and S2 for individual plots of the pyramidal parameter space for LV food chains of different lengths, up to six trophic levels). There are indeed parameter combinations that give rise to Eltonian pyramids in both model situations (i.e. with and without DDCM). However, in LV models *without* DDCM, the parameter space allowing for pyramidality in chains of different lengths does not seem to overlap (since only unhatched areas with different shades of green can be found, corresponding to pyramidality in food chains with either two, three or four trophic levels, Fig. 1A). With DDCM instead, there is considerable overlap in the pyramidal parameter space for chains of different lengths (here, single and double hatched areas), so that it is possible for food chains with two AND three trophic levels, as well as two, three AND four trophic levels to be pyramidal simultaneously (i.e. for the same parameter combinations; diagonally hatched, intermediately green sector and double hatched, yellow sector respectively, Fig. 1B). In fact, the pyramidal parameter space for one chain length (e.g. three trophic levels) is now to a large extent found within the pyramidal parameter space of the shorter chain (i.e. with two trophic levels). Changing the value of the mortality rates of the consumers does not qualitatively alter the results (see Figs S3, S4 for results where consumer mortality rates are one magnitude greater and smaller respectively than in Fig. 1) and the same is true for other intervals of *a*_*ij*_ and *a*_*ji*_ (see analytical results below for general support of this). For example, increasing *b*_*i*_ (*i* ≥ 2), i.e. decreasing the consumers mortality rates (absolute values) only has the effect of increasing the parameter space that allows pyramidality in the chains. The parameter space for chains with different number of trophic levels still cannot be seen to overlap without density-dependent consumer mortality (Fig. S4).

Above, no assumption has been made on any relationship between the consumer and resource interaction strengths. However, it is not unusual in modelling studies to assume that the effect of a resource on a consumer (*a*_*ji*_) is a proportion of the effect of the consumer on the resource (*a*_*ji*_) (see methods). This alternative, i.e. *a*_*ji*_ = *γ* × *a*_*ji*_, has also been analysed here. For *γ* = 0.1 there is no value of the consumer interaction strength within the interval ${a}_{i,i+1}=[{10}^{6}\,{10}^{-6}]$, that allow LV food chains with different number of trophic levels to be pyramidal simultaneously (i.e. for the same value of *a*_*i,i*+1_) *without* DDCM (Fig. 1C). However, with DDCM there is a narrow region of values for *a*_*i,i*+1_ where this is possible (Fig. 1D). These results hold also for other values of *γ*, thus showing that irrespective if there is a relationship between the consumer and resource interaction strengths or not, LV food chains with different number of trophic levels cannot be pyramidal simultaneously (i.e. for the same value of *a*_*i,i*+1_) without DDCM, while with DDCM this is possible.

### Changing interaction strengths and mortality rates with trophic height

As a more complex but potentially more realistic scenario, assume that (*a*) consumer mortality rates are negatively related to the trophic position since (*i*) mortality rate is a decreasing function of body size and (*ii*) body size is an increasing function of trophic position^48, 49^, (*b*) the resource interaction strengths increase with trophic position since (*i*) resource interaction strengths could be negatively related to the consumer resource body mass ratio and (*ii*) there is a tendency for the consumer resource body mass ratio to decrease with increasing trophic position in many real food webs^48, 49^, and (*c*) the consumer interaction strengths decrease with trophic position since (*i*) consumer interaction strengths could be positively related to the consumer resource body mass ratio and (*ii*) there is a tendency for the consumer resource body mass ratio to decrease with increasing trophic position in many real food webs. These assumptions can be formalized mathematically to describe how interaction strengths and mortality rates change with trophic position (see Methods). Figure 2A,B show the combinations of the parameters *a*_12_ and *a*_21_ that under these assumptions, allow pyramidality in LV food chains with two, three and four trophic levels, without (Fig. 2A) and with (Fig. 2B) DDCM respectively. Contrary to Fig. 1A, where the pyramidal parameter space could not be seen to overlap, it is now possible to find a small region that allow chains with two, three and four trophic levels to be pyramidal simultaneously, even without DDCM (Fig. 2A). With DDCM instead, there is an even larger area of pyramidal parameter space where chains of different length can be pyramidal simultaneously, when interaction strengths and mortality rates change with trophic position (Fig. 2B) compared to if they are not (Fig. 1B). (See Figs S5 and S6 for individual plots of the pyramidal parameter space for LV food chains of different lengths, up to six trophic levels, and Figs S7 and S8 for results where consumer mortality rates are one magnitude greater and smaller respectively than in Fig. 2.) The present scenario with changing interaction strengths and mortality rates with trophic height has also been analysed under the alternative that consumer and resource interaction strengths are related via the ecological efficiency (i.e. *a*_*ji*_ = *γ* × *a*_*ji*_). As above, results are qualitatively the same: without DDCM it is difficult for LV food chains with different number of trophic levels to be pyramidal simultaneously (i.e. for the same value of *a*_*i,i*+1_, Fig. 2C), while this is possible for many food chain lengths with DDCM (Fig. 2D).

### Analytical results

In Notes S2–S4 analytical results are derived for LV food chains with two, three and four trophic levels, to support the findings above. Note S2 shows the trophic level equilibrium densities as functions of the interaction coefficients, *a*_*ij*_, and mortality rates, *b*_*i*_, of the model (1), and general conditions for feasibility and pyramidality. Based on this, specific conditions for pyramidality are derived under the same two scenarios as above: (*i*) constant interaction strengths and mortality rates across trophic levels (Note S3) and (*ii*) changing interaction strengths and mortality rates with trophic position (Note S4). These show that with constant mortality rates and interaction strengths across trophic levels and without DDCM the conditions for pyramidality in food chains with different numbers of trophic levels (Eqs S3.5–S3.7) are not compatible (Note S3), thus proving that the parameter spaces allowing for pyramidality in Fig. 1A really do not overlap for chains with different numbers of trophic levels. With DDCM however, the conditions for pyramidality (Eqs S3.5–S3.7) are more easily fulfilled and it is possible for food chains with different numbers of trophic levels to be pyramidal simultaneously (i.e. for the same set of parameter values), thus supporting the partly overlapping pyramidal parameter spaces for food chains with different numbers of trophic levels in Fig. 1B. Finally, allowing interaction strengths and mortality rates to change with trophic position, it is proven that both decreasing mortality rates and increasing resource interaction strengths with trophic position promotes the existence of pyramidality in LV food chains (Note S4). To summarize, the graphical results in Figs 1 and 2 are all supported by the analytical results.

### Randomly drawn interaction strengths and mortality rates

The analyses above have been complemented by simulations where the mortality rates and interaction strengths were drawn at random from certain intervals (see Note S5 and Figs S9–S11). In line with the results reported above I find the proportion of such model food chains that have a pyramidal pattern in the distribution of abundance to be far greater with DDCM than without (Table S1). Also, the probability that such a food chain is pyramidal both before and after a deletion of the top trophic level (i.e. possessing the property of ‘deletion robust pyramidality’) is significantly higher with DDCM. Furthermore, although drawing interaction strengths at random from the same interval for every trophic level, I find the realized mean predator interaction strength (mean|*a*_*i,i*+1_|) to be significantly negatively correlated and mean prey interaction strength (mean(*a*_*i*+1*,i*_)) positively correlated to the length of the food chains (Table S2; Fig. S10) in feasible and pyramidal food chains as well as model chains that possess the property of deletion robust pyramidality. Thus, the criteria of feasibility, pyramidality and deletion robust pyramidality seems to select replicates from the random set of model chains that have similar characteristics (i.e. changing interaction strengths and mortality rates with trophic height) as the model chains that in Fig. 1 were shown to increase the number of trophic levels that could be added to or lost from a model chain, while still retaining the property of pyramidality.

## Discussion

The predictions on (relative) trophic level abundances in Lotka-Volterra food chains under different model assumptions have been investigated here. First, it is pointed out that empirical data indicate that trophic pyramids of numerical abundance are the norm in real communities and shown that this is supported by metabolic arguments. The latter furthermore suggests that this pyramidal pattern should not be disrupted if the number of trophic levels change. This makes it interesting to analyse if community models also are able to generate a ‘consistent pyramidal pattern’ of trophic level equilibrium abundances, such as that the pyramidal pattern is not disrupted if the number of trophic levels change. In other words, not only if there are parameter combinations of food chain models that render a particular food chain (of length *n*) pyramidal (which is trivial), but whether such food chains stay pyramidal if trophic levels are added or removed. Second, it is shown that assuming constant resource interaction strengths as well as mortality rates across trophic levels, absence of density dependent consumer mortality (DDCM) makes it impossible for LV food chains to pass this test. That is, if a model chain with *n* number of trophic levels is pyramidal, it is not for *n* − 1 or *n *+ 1 trophic levels for that parameter setting. This means that there is one number of trophic levels only that generates an Eltonian pyramid for each combination of *b*_2_ and *a*_21_, without DDCM, and would imply that a community that is pyramidal could never lose or gain a trophic level and remain pyramidal (unless the interaction strengths are modified during this process). By including DDCM into LV models this clearly unrealistic prediction can be avoided, since it is now possible for LV food chains of adjacent lengths to be pyramidal. Thus, under the assumption of constant resource interaction strengths as well as mortality rates across trophic levels, DDCM needs to be incorporated into LV models for them to provide realistic predictions on (relative) trophic level abundances.

In reality however, constant mortality rates and interaction strengths may be an unrealistic assumption. Instead, real food chains may be characterized by a specific patterning of both these parameters^15, 29, 42, 46^. Next, and in line with this, it is shown that biologically motivated relationships between model parameters and trophic position can make it somewhat easier for food chains of adjacent lengths to be pyramidal, even without DDCM. For example, both mortality rates and interaction strengths may change with trophic position in ecological communities. In particular, mortality rates are often considered to decrease with body size and trophic interaction strengths have been argued to be affected by consumer resource body mass ratios^26, 47^. That is, since the energetical value of a resource to a consumer increases with the size of the resource, relative to the consumer, the resource interaction strength should be positively related to the resource-consumer body mass ratio^42, 50^. Furthermore, there is a tendency for the consumer resource body mass ratio to decrease with increasing trophic height in many real food webs^48, 49^ which would mean that the resource interaction strengths could increase with trophic position. Taking this into account by assuming that consumer mortality rates are negatively, and resource interaction strengths positively related to trophic position it is now possible to find small regions in parameter space that allow chains with two, three and four trophic levels to be pyramidal simultaneously, even without DDCM (Fig. 2A). Thus, this improves on previous results^10^ by showing that using biological and ecological arguments when choosing the relative magnitudes of the resource interaction strengths and consumer mortality rates, can make it possible (but still difficult) for LV food chains without DDCM, to have a pyramidal pattern of abundance for more than one chain length. This could either mean that Eltonian pyramids are unlikely to occur in the real world without a specific set of interaction strengths and consumer mortality rates, or that LV food chains without DDCM are unrealistic representations of real food chains. Results from combining DDCM with decreasing mortality rates and changing interaction strengths with increasing trophic position argues for the latter, since the region parameter space that allow for pyramidality for more than one chain length now is very large (Fig. 2B). Relating the model parameters to observed body size distributions has in other studies been shown to increase the resilience of LV food chains^42^ as well as food web stability^16^. Hence, different ecological aspects, such as resilience, stability and probability for pyramidality, all seem to be improved simply by relating model parameters to body size.

The analyses and discussion this far have (at least implicitly) assumed strict pyramidality, i.e. a regular decrease in equilibrium abundances, from the primary trophic level to the top level. Since there are well-known exceptions to this, such as the number of herbivorous insects exceeding the number of trees in terrestrial forest ecosystems, or inverted biomass pyramids^7^ the results presented here might not seem very general. However, most non-compliances to strict pyramidality involve the abundance of the second trophic level exceeding that of the first trophic level (although other exceptions are possible for the distribution of biomass^1^). This case can easily be accommodated in the present study by ignoring the abundance of the second trophic level relative to the first and analysing where non-strict pyramidality (i.e. a regular decrease in equilibrium abundances, from the second trophic level to the top level) is possible. The result of this relaxation does not qualitatively alter the results presented here. For example, Eq. S3.9 combines the conditions ensuring that *N*_2_^*^ > *N*_3_^*^ in a three and four level food chain and does not rely on any specific relation between *N*_1_^*^ and *N*_2_^*^. This condition thus puts the same pyramidal restrictions on parameter values in any system regardless of the relationship between the abundance of the first and second trophic level. More generally, with constant mortality rates and interaction strengths across trophic levels and without DDCM it is still not possible for LV food chains of adjacent lengths to be (non-strict) pyramidal, while if including DDCM and/or allowing mortality rates and interaction strengths to change with trophic position this becomes possible. Thus, the results presented here are not sensitive to the kind of system chosen to model (e.g. to the size of the primary producer relative to the primary consumer).

A relevant question is what mechanism are involved that could make it easier for LV food chains with DDCM to show a pyramidal pattern of trophic level equilibrium abundances. It is well-known that under donor-controlled dynamics, where each level exploits all the energy that it gets from below, but does not affect the level below (i.e. no top-down limitation of prey by predator), metabolic constraints predict that the equilibrium numerical abundance should decrease with increasing trophic level, thus producing a pyramid of numbers (See Note S1). It could therefore be hypothesized that the role of DDCM is to push the dynamics from top-down towards more bottom-up control (i.e. in the direction of donor-control). In other words, the more a consumer trophic level is affected by DDCM, the less likely it is to exert strong top-down control on the level below, as well as being regulated by the level above, and thus perturb the ‘default’ donor-control pyramid. To study this, we could analyse if parameter combinations that allow for pyramidality coincide with parameter combinations where the effect of self-limitation on a trophic level is greater than that of predation from the trophic level above (indicating that bottom-up control could be stronger than top-down). Accordingly, I analysed this possibility, for every combination of *a*_21_ and *a*_12_ in Fig. 1B and for food chains with three to six trophic levels, by calculating the metric

2 $$\min ({a}_{i,i}{N}_{i}^{\ast }/{a}_{i,i+1}{N}_{i+1}^{\ast })\,(i > 1)$$

A ratio greater than unity means that the effect of self-limitation on every consumer trophic levels is stronger that that exerted by predation from the trophic level above (implying that every trophic level is more affected by bottom-up control than top-down). Figure S12 show the parameter combination of *a*_21_ and *a*_12_, where this index is greater than unity, for food chains with three to six trophic levels. By comparing this to the parameter combination of *a*_21_ and *a*_12_ allowing for pyramidality in Fig. S2B–E, it is obvious that the hypothesis on the role of DDCM as promoting bottom-up control, only gain partial support. The areas in Fig. S2B–E (showing pyramidality) only partly overlap with those in Fig. S12A–D (showing where self-limitation is stronger top-down control). More specifically, the lower boundary on *a*_21_ allowing for pyramidality (Fig. S2B–E) seem to overlap with that where self-limitation is stronger top-down control (Fig. S12A–D), while there is no such correspondence for the upper boundary on *a*_21_. Thus, the positive effect of DDCM on pyramidality in LV food chains can only partly be attributed to pushing the dynamics from top-down towards more bottom-up control.

My analyses of conditions for pyramidality above are based on generalized Lotka-Volterra models with linear functional responses. Such models have a long history in ecology, but have also been used in many recent studies, for example to explore the dynamics of food webs and their response to different stressors^51–54^. Admittedly, many alternatives for modelling trophic dynamics exist (e.g., using models with non-linear functional responses^55, 56^) that may more realistically capture the foraging success of individual consumers across large ranges of prey densities. However, little empirical support exists for the superiority of these alternatives over the simpler model used here, for the population response over more moderate ranges of prey densities. Indeed, Novak^57^ estimated interaction strengths empirically and analysed the potential non-linearity of observed functional responses, finding that models assuming linear functional responses actually performed better than others. Novak’s study contributes to a growing literature suggesting that “trophic interactions are approximately linear in the range of mean prey densities actually observed in nature, especially in multispecies settings”^57^. Furthermore, some theoretical studies have compared model results based on linear and non-linear functional responses, and found that relationships between structure and stability and general patterns in the response of food webs to species loss were similar for the two scenarios^58–60^. Overall, these studies suggest that results, based on models with linear functional responses, although not accurately describing individual level relationships, actually have something to say about population level phenomena. The validity of this interpretation for pyramidality in food chains is here supported by complementary simulation results on the presence of Eltonian pyramids in LV food chains, where consumers have a type 2 functional response. With a weak type 2 functional response (parameter *c*_*j*_ small, see Fig. S13) results are practically identical to those in Fig. 1, so that without DDCM it is impossible for LV food chains with different number of trophic levels to be pyramidal simultaneously (Figs S13, S14 As the saturation effect in the functional response becomes stronger (increasing *c*_*j*_) some overlap appear in the pyramidal parameter space for food chains of different lengths, but it is still impossible to find any region that allow chains with two, three and four trophic levels to be pyramidal simultaneously, without DDCM. In other words, although results are somewhat quantitatively modified with a non-linear functional response, results are qualitatively unchanged, thus reinforcing the conclusion that Eltonian pyramids are difficult to reproduce in LV food chain models without density-dependent mortality in the consumers.

The analytical analyses (Notes S2–S4) are focused on food chains of length two to four. Apart from reasons of tractability, the rationale for this is that in most real systems the majority of food chains do not have more than four ‘true’ trophic levels. Where the number of levels appear to exceed four, this often involves omnivory where the higher trophic levels feed on several lower levels. However, similar analyses of the conditions assuring pyramidality, in food chains of different lengths that have been done here (e.g. Eqs S3.8–S3.10) show that under assumption Eq. (S3.12), simultaneous pyramidality in food chains of length four, five and six is impossible (results not shown here). Hence, the results presented here are valid for longer chains as well and thus general.

The distribution of numerical and biomass abundances within a community is not merely a neat graphical summary of the trophic structure of a system, but also conveys information on patterns of flow, turnover and transfer efficiency of energy, as well as mechanisms regulating populations. Since a fundamental goal in ecology is to understand the processes that structure communities and give them their functional characteristics, it is obvious that increased understanding of the mechanisms that shape the trophic stack of abundances has a key role to play in this quest. Community models could be a useful theoretical tool to analyse these mechanisms, allowing population dynamics to be played out within metabolic constraints acting on populations, and studying how bottom-up and top-down effects combine to affect the distribution of abundances. However, before this goal can be reached, discrepancies between ecological data and theory needs to be identified and bridged. Here, I have identified a potential weakness in traditional LV predator-prey models and suggested how this can be overcome. Specifically, the implications for future studies, using the LV food chain approach, is that density-dependent consumer mortality should be considered and other model parameters should, as far as possible, be related to organism body sizes, in order for the model to provide a realistic representation of real ecological communities. More generally, with this it will be possible to, with more confidence, study the mechanisms that shape the trophic stack of abundances. For example, how anthropogenic pressure on species and ecosystems may affect their structure and function, or more thoroughly analyse mechanisms that can lead to ‘top-heaviness’ in the distribution of biomass (where the abundance of a consumer approaches or even exceeds that of its resource). The latter is relevant since inverted pyramids of biomass are known to exist and could be more frequent than until now believed.

To conclude, empirical data and metabolic reasoning indicate that trophic pyramids of numerical abundance should be the norm in real communities, while I here show that a consistent pyramidal pattern is difficult to obtain from simple LV models without density-dependent mortality in the consumers, unless biologically plausible relationships between body size, mortality rate and interaction strengths are taken into account. By addressing this weakness, LV models can continue to be a fruitful tool for providing insights into the mechanisms that affect ecological structure and function.

## Methods

In this paper, parameter combinations that allow for a pyramidal pattern of abundance, in LV food chains of the form described by Eq. (1), were analysed under different assumptions. First, consumer mortality rates (*b*_*i*_, *i* ≥ 2) as well as consumer and resource interaction strengths (the *a*_*ij*_’s and the *a*_*ji*_’s, *j* = *i* + 1) were assumed to be constant (the same) within a food chain and without any relationship between consumer and resource interaction strengths. That is:

3 $$\{\begin{array}{rcl}{a}_{i,i+1} & = & {a}_{12}(i=2,\mathrm{..}.\,n-1)\\ {a}_{i+1,i} & = & {a}_{21}(i=2,\mathrm{..}.\,n-1)\end{array}$$

where *i* refers to the trophic position of species *i* in the chain. Since the choices of units for area (used to measure abundance) and time are arbitrary, the intrinsic growth rate of the producer trophic level (*b*_1_) and the intraspecific interaction term (*a*_11_) were both set to unity. Consequently, all other parameters were scaled relative to *b*_1_ and *a*_11_. I furthermore assumed a constant (density independent) consumer mortality rate (*b*_*i*_ = 0.0001, *i* ≥ 2), i.e., the same for every consumer trophic level. Under these assumptions, and for every combination of parameters *a*_12_ and *a*_21_ within the interval (using a log_10_-linear grid of 1000 × 1000):

4 $$\{\begin{array}{l}{a}_{i,i+1}=[{10}^{6}\quad {10}^{-6}]\,\\ {a}_{i+1,i}=[{10}^{-3}\quad {10}^{3}]\end{array}$$

it was first analysed if LV food chains of lengths 2, 3 and 4, respectively, produced a pattern of equilibrium abundances that were both feasible (positive abundance for every trophic level) and pyramidal (decreasing abundance with increasing trophic position), (*i*) without density dependent consumer mortality (DDCM) (*a*_*ii*_ = 0, *i* ≥ 2,) as well as (*ii*) with DDCM (*a*_*ii*_ = 1, *i* ≥ 2) respectively (see Fig. 1A,B).

Second, it was assumed that the effect of a resource on a consumer (*a*_*ji*_) was a proportion of the effect of the consumer on the resource (*a*_*ji*_) so that:

5 $${a}_{i+1,i}=\gamma \times {a}_{i,i+1}$$

All else above unchanged, this alternative was analysed for *γ* = 0.1 (Fig. 1C,D).

Third, it was assumed that (*a*) consumer mortality rates are negatively related to the trophic position, (*b*) the resource interaction strengths increase with trophic position, and (*c*) the consumer interaction strengths decrease with trophic position according to the following relationships:

6 $$\{\begin{array}{ll}{a}_{i,i+1}={k}_{cons}^{i-1}{a}_{1,2} & (i=1,\mathrm{..}.\,n-1)\\ {a}_{i+1,i}={k}_{res}^{i-1}{a}_{2,1} & (i=1,\mathrm{..}.\,n-1)\\ {b}_{i}={k}_{b}^{i-2}{b}_{2} & (i=2,\mathrm{..}.\,n-1)\end{array}$$

where *k*_*cons*_ < 1, *k*_*res*_ > 1 and *k*_*b*_ < 1 describe how interaction strengths and mortality rates change with trophic position. For *k*_*b*_ = 0.1, *k*_*res*_ = 2 and *k*_*cons*_ = 0.5, it was analysed as above if LV food chains of lengths 2, 3 and 4, respectively, produced a pattern of equilibrium abundances that were both feasible (positive abundance for every trophic level) and pyramidal (decreasing abundance with increasing trophic position), (*i*) without density dependent consumer mortality (DDCM) (*a*_*ii*_ = 0, *i* > 2), as well as (*ii*) with DDCM (*a*_*ii*_ = 1, *i*  > 2) respectively (see Fig. 2A,B)

Fourth, the third scenario with changing interaction strengths and mortality rates with trophic height (Eq. 5) was also studied under the alternative that consumer and resource interaction strengths are related via the ecological efficiency (Eq. 4). All else unchanged, this alternative was analysed for *γ* = 0.1 (Fig. 2C,D).

Fifth, analyses of the deterministic scenarios one and three above were complemented by simulations where model parameters (mortality rates and interaction strengths) were drawn at random from specific intervals, instead of being assigned certain values (see Note S5).

Finally, analytical results were obtained by deriving expressions for the equilibrium abundances in two, three and four trophic level LV food chains, and from this developing conditions for feasibility as well as pyramidality (Notes S2–S4).

### Data availability

The datasets generated and analysed during the current study are available from the corresponding author on reasonable request.

## Electronic supplementary material

Supplementary Information
