# Supplementary material for: Conditions for Eltonian Pyramids in Lotka-Volterra Food Chains
Source: Sci Rep. 2017 Sep 7;7:10912. doi: 10.1038/s41598-017-11204-1 (PMC5589755; doi:10.1038/s41598-017-11204-1)
Supplement: Supplementary file 1 — Supplementary Information [file 41598_2017_11204_MOESM1_ESM.pdf]

Online Supplementary Information for "Conditions for Eltonian Pyramids in Lotka-Volterra Food Chains" by T. Jonsson

This paper includes the following supplementary information as supporting material:

Note S1: Derivations of metabolic constraints on the shape of ecological pyramids (with one in-Note figure I).

Note S2: Analytical derivations of equilibrium densities and general conditions for pyramidality in LV food chains

Note S3: Analytical derivations of conditions for pyramidality in LV food chains assuming constant mortality rates and no density dependent consumer mortality

Note S4: Analytical derivations of conditions for pyramidality in LV food chains allowing interaction strengths and mortality rates to change with trophic position

Note S5: Results of simulations of LV food chains with randomly drawn interaction strengths and mortality rates

Figure S1: Parameter combinations of resource and consumer interaction strengths allowing for pyramidality in Lotka-Volterra food chains with different number of trophic levels assuming constant mortality rates and no density dependent consumer mortality.

Figure S2: Same as Fig. S1 except for inclusion of density dependent consumer mortality.

Figure S3: The presence of Eltonian pyramids in Lotka-Volterra (LV) food chains with different number of trophic levels, under the assumption of constant consumer mortalities and interaction strengths with trophic position.

Figure S4: Same as Fig. S3 except for $b_i = 0.00001\ (i \geq 2)$.

Figure S5: Parameter combinations of resource and consumer interaction strengths allowing for pyramidality in Lotka-Volterra food chains with different number of trophic levels assuming no density dependent consumer mortality, and interaction strengths and mortality rates that change with trophic position.

Figure S6: Same as Fig. S3 except for inclusion of density dependent consumer mortality.

Figure S7: The presence of Eltonian pyramids in Lotka-Volterra (LV) food chains with different number of trophic levels, assuming interaction strengths and mortality rates that change with trophic position.

Figure S8: Same as Fig. S7 except for $b_i = 0.00001\ (i \geq 2)$.

Figure S9: Proportion of Lotka-Volterra food chains with randomly drawn interaction strengths and mortality rates that are species-deletion pyramidal.

Figure S10: Mean consumer and resource interaction strengths for Lotka-Volterra food chains with randomly drawn interaction strengths and mortality rates that are feasible, pyramidal and species-deletion pyramidal

Figure S11: Mean consumer and resource interaction strengths at different trophic positions in Lotka-Volterra food chains with randomly drawn interaction strengths and mortality rates that are feasible, pyramidal and species-deletion pyramidal.

Figure S12: Top-down vs. bottom-up control in Lotka-Volterra food chains with different number of trophic levels, with density-dependent consumer mortality.

Figure S13: The presence of Eltonian pyramids in Lotka-Volterra (LV) food chains with different number of trophic levels, where consumers have a type 2 functional response, assuming constant mortality rates and no density dependent consumer mortality.

Figure S14: Same as Fig. S12 except for inclusion of density dependent consumer mortality.

Table S1: Effect of density dependent consumer mortality on the pyramid of numbers in Lotka-Volterra food chains with randomly drawn interaction strengths and mortality rates.

Table S2: Correlation between number of trophic levels and mean consumer and resource interaction strengths in Lotka-Volterra food chains

Table S3: Correlation between trophic position and mean consumer and resource interaction strengths in Lotka-Volterra food chains

NOTE S1 – METABOLIC CONSTRAINTS ON THE SHAPE OF ELTONIAN PYRAMIDS

In this note I analyze metabolic constraints on the shape of Eltonian pyramids by deriving relationships for the abundance of a trophic level, relative to that of the level below it. Three assumptions on the amount of energy available by each trophic level are used as alternative starting points. First, as a simple null-hypothesis, I assume that all trophic levels have access to and use the same amount of energy, per unit time. Second, I take a "bottom-up control" approach and assume that every trophic level exploits all (or the same proportion of) the available energy at the level below, but does not affect the level below (i.e. no top-down limitation of resources by consumers). This means that energy availability, due to ecological efficiencies, must decrease with every trophic level. Third, I relax any assumption on how energy use may change with trophic level, by acknowledging that consumers can reduce the abundance of their resources and that production of new biomass tends to vary non-linearly with abundance. As a result, this final analysis looks at the consumer abundance that can be supported by different levels of resource abundance, via differences in resource production. It must be stressed however, that nowhere in this note are the abundances of trophic levels set dynamically by both bottom-up and top-down mechanisms. Here instead, constraints on the shape of Eltonian pyramids are derived, using simple metabolic arguments, which predictions from the dynamical model defined by Eq. 1 can be compared with.

To set the stage, start by considering the processing of energy of organisms. The per individual resource (energy) use, *e*, is assumed to be proportional to the metabolic rate of the individual, *R*, which is allometrically related to body size, *W*, (Gillooly *et al.* 2001, Brown *et al.* 2004):

$$e \propto R \propto W^{\beta} \qquad \text{(S1.1)}$$

where β is the metabolic exponent, often claimed to approximate ¾ (Kleiber 1947, Hemmingsen 1950, Brown *et al.* 2004). This implies that the per population resource (energy) use, *E*, is

$$E \propto N \times R \propto N \times \overline{W}^{\beta} \qquad \text{(S1.2)}$$

Where $\overline{W}$ is the average body size of a population. Next, apply this framework to a trophic network by considering a linear trophic chain describing the energy flow between trophic levels in a community. As described by Teramoto (1993), for example, any food web structure can, from an energy flow perspective, be collapsed to such a chain, by 'unfolding its energy-flow network' (i.e. partitioning the biomass of each species/node to different trophic levels according to the path lengths of its origin). Let ε_T denote the energy available (per unit time) to trophic level *T*, and E_T the total energy consumption (turnover) rate at trophic level *T*. Furthermore, N_T, $\overline{R}_T$ and $\overline{W}_T$ denotes the total abundance, average metabolic rate and average body size of individuals, at trophic level *T*, respectively.

Now, assume initially that all trophic levels (populations) at, or near equilibrium, are independent of each other and have access to and use the same amount of energy, per unit

time (this clearly unrealistic assumption will be relaxed below). This means that $\varepsilon_T = E_T = \varepsilon_{T+1} = E_{T+1}$ and implies:

$$N_T \times \overline{R}_T = N_{T+1} \times \overline{R}_{T+1} \quad \Leftrightarrow \quad N_T \times \overline{W}_T^{\beta} = N_{T+1} \times \overline{W}_{T+1}^{\beta} \qquad \text{(S1.3)}$$

Rearranging and using ρ_T to denote the average consumer-resource body mass ratio, $\overline{W}_{T+1} / \overline{W}_T$, between trophic levels T+1 and T, results in:

$$\frac{N_{T+1}}{N_T} = \left(\frac{\overline{W}_T}{\overline{W}_{T+1}}\right)^{\beta} = \left(\frac{1}{\rho_T}\right)^{\beta} = \rho_T^{\,-\beta} \qquad \text{(S1.4)}$$

Although there are well-known examples of consumers being smaller than their resources (e.g. pack hunters such as wolves or hyenas, or a carabid beetle attacking an earthworm or snail), true predators (i.e. consumers that are not parasites, parasitoids or pathogens) tend to be larger than their resource (so that $\rho > 1$, Cohen *et al.* 1993, Brose *et al.* 2006). Since $0 < \beta < 1$, Eq. S1.4 implies that the equilibrium numerical abundance ratio between adjacent trophic levels is smaller than unity, i.e. $N_{T+1} / N_T < 1$. In other words, the equilibrium numerical abundance is predicted to decrease with increasing trophic level, producing a pyramid of numbers. The greater the difference in body size between individuals on adjacent trophic levels (i.e. the greater the consumer-resource body mass ratio), the greater the difference in numbers (equilibrium numerical abundance) between adjacent trophic levels. Furthermore, since biomass is the product of body mass and numerical abundance, the following relationship describes the ratio in biomass abundance between adjacent trophic levels:

$$\frac{B_{T+1}}{B_T} = \frac{N_{T+1} \times \overline{W}_{T+1}}{N_T \times \overline{W}_T} = \left(\frac{\overline{W}_T}{\overline{W}_{T+1}}\right)^{\beta} \times \frac{\overline{W}_{T+1}}{\overline{W}_T} = \left(\frac{1}{\rho_T}\right)^{\beta} \times \rho_T = \rho_T^{\,1-\beta} \qquad \text{(S1.5)}$$

With $\rho_T > 1$ and $0 < \beta < 1$, this implies that the equilibrium biomass abundance ratio between adjacent trophic levels is greater than unity, i.e. $B_{T+1} / B_T > 1$ (and increases slowly with increasing consumer-resource body mass ratio). In other words, the equilibrium biomass abundance is predicted to increase with increasing trophic level, producing an inverted pyramid of biomass.

Next, introduce some realism by assuming that every trophic level is dependent on the level below, but either do not affect its abundance (i.e. strict donor control) or reduces the level below to the same extent, so that every trophic level exploits a constant proportion of the available energy at the level below. With ecological *in*efficiencies ($\gamma < 1$) this means that energy availability (ε) and use (E) should decrease with trophic level. With γ_T denoting the ecological efficiency in energy transfer between trophic levels T+1 and T, (so that $E_{T+1} = \gamma_T \times E_T$) then:

$$E_{T+1} = \gamma_T \times E_T \quad \Leftrightarrow \quad \gamma_T \times N_T \times R_T = N_{T+1} \times R_{T+1} \quad \Leftrightarrow$$

$$\gamma_T \times N_T \times \overline{W}_T^{\beta} = N_{T+1} \times \overline{W}_T^{\beta} \quad \Rightarrow \quad \frac{N_{T+1}}{N_T} = \gamma_T \times \left(\frac{\overline{W}_T}{\overline{W}_{T+1}} \right)^{\beta} = \qquad \text{(S1.6)}$$

$$= \gamma_T \times \left(\frac{1}{\rho_T} \right)^{\beta} = \gamma_T \times \rho_T^{\;-\beta}$$

Again, (as in Eq. S1.4) the equilibrium numerical abundance is predicted to decrease with increasing trophic level, producing a pyramid of numbers, but with an even greater difference in numbers (equilibrium numerical abundance) between adjacent trophic levels if $\gamma_T < 1$ (compared to $\gamma_T = 1$). Finally for biomass abundance:

$$\frac{B_{T+1}}{B_T} = \frac{N_{T+1} \times \overline{W}_{T+1}}{N_T \times \overline{W}_T} = \gamma_T \times \left(\frac{\overline{W}_T}{\overline{W}_{T+1}} \right)^{\beta} \times \frac{\overline{W}_{T+1}}{\overline{W}_T} = \gamma_T \times \left(\frac{1}{\rho_T} \right)^{\beta} \times \rho_T = \gamma_T \times \rho_T^{\;1-\beta} \qquad \text{(S1.7)}$$

Whether biomass abundance decreases or increases with increasing trophic level depends on the balance between γ_T and ρ_T. For example, $\beta = ¾$ and $\gamma_T = 0.1$ will ensure that ${\overline{B}_{T+1}}/{B_T} < 1$ (instead of ${\overline{B}_{T+1}}/{B_T} > 1$ as in Eq. S1.5) as long as $\rho_T < 10^4$. Thus, with $\gamma_T << 1$ equilibrium biomass abundance is predicted to decrease with increasing trophic level, producing a regular pyramid of biomass (as long as γ is small and the consumer-resource body mass ratio not very large). More specifically, γ needs to be smaller than $\rho^{\beta-1}$ to produce pyramids of biomass. To summarize, this illustrates the principle that the relationship between consumer and resource abundance (and thus the shape of Eltonian pyramids) in a bottom-up controlled situation depends on (*i*) the ratio (ρ_T) in average body size ($\overline{W}$) between organisms on adjacent trophic levels and (*ii*) the efficiency of energy transfer (γ_T) between trophic levels.

However, energy available to trophic level T+1 (ε_{T+1}) need not be proportional to the turnover rate of energy on the level below (E_T), since with increasing abundance of a trophic level more and more energy may go to waste as maintenance costs, intraspecific mortality etc. (and thus not be available to the next trophic level). Instead, energy available to trophic level T+1 could be proportional to the production rate of new biomass (Π) at the level below (i.e. $\varepsilon_{T+1} \propto \Pi_T$). Contrary to turnover of energy (which increases allometrically with abundance), production of new biomass tends to be a hump-shaped function of abundance in many populations, first increasing and peaking at an intermediate abundance, before decreasing with further increase in abundance. Since consumers may affect the abundance of their resources, and thus the energy available to themselves, via the production rate of new biomass, it is relevant to study the abundance at trophic level T+1 that can be supported by different levels of production of new biomass at level T, and if there is any range of abundance of trophic level T that in theory could support a higher abundance on level T+1 (so that $N_{T+1} > N_T$). To analyze the metabolic constraints set by this scenario (without allowing the abundances of trophic levels to be set dynamically by both bottom-up and top-down mechanisms), assume

that the energy need of trophic level T+1 (E_{T+1}, J×day^{-1}) increases allometrically with body size and linearly with abundance as $E_{T+1} = \delta_1 \overline{W}_{T+1}^{\beta} N_{T+1}$, where $\overline{W}$ is measured in g and δ_1 is a scaling (normalization) constant (describing the field metabolic rate, J×day^{-1}, of an individual with body size 1 g). Assume furthermore, for simplicity that trophic level T grows logistically, so that its production of new biomass can be described as:

$$\Pi_T = \overline{W}_T r_T N_T \left(1 - \frac{N_T}{K_T}\right) \qquad \text{(S1.8)}$$

where r_T is the intrinsic growth rate (day^{-1}) and K_T the carrying capacity. This can be converted to energy potentially available to trophic level T+1 (ε_{T+1}, J×day^{-1}) as:

$$\varepsilon_{T+1} = \varphi \Pi_T \delta_2 \qquad \text{(S1.9)}$$

where δ_2 is the energy content of 1 g (fresh weight) of biomass and φ is the combined ingestion-assimilation efficiency. The latter represents the proportion of each unit of biomass removed from trophic level T by consumers on level T+1 that is actually ingested and assimilated (e.g. many carnivores only consume part of its kill). The intrinsic growth rate r_T increases allometrically with body size across a large range of organismal body sizes (Fenchel 1974, Blueweiss et al. 1978, Brown et al. 2004) as $r_T = \delta_3 \overline{W}_T^{\beta-1}$ where δ_3 is a scaling (normalization) constant (describing the intrinsic growth rate of an individual with body size 1 g). To see what numerical abundance of trophic level T+1 that the production of new biomass at trophic level T can support, set $E_{T+1} = \varepsilon_{T+1}$, which results in:

$$\delta_1 \overline{W}_{T+1}^{\beta} N_{T+1} = \varphi \overline{W}_T \delta_3 \overline{W}_T^{\beta-1} N_T \left(1 - \frac{N_T}{K_T}\right)\delta_2 \Leftrightarrow$$
$$N_{T+1} = \varphi \left(\frac{\overline{W}_T}{\overline{W}_{T+1}}\right)^{\beta} \frac{\delta_2 \delta_3}{\delta_1} N_T \left(1 - \frac{N_T}{K_T}\right) \qquad \text{(S1.10)}$$

For biomass abundance the same exercise results in:

$$B_{T+1} = \varphi \left(\frac{\overline{W}_{T+1}}{\overline{W}_T}\right)^{1-\beta} \frac{\delta_2 \delta_3}{\delta_1} B_T \left(1 - \frac{N_T}{K_T}\right) \qquad \text{(S1.11)}$$

From this it is evident that the numerical as well as biomass abundance of trophic level T+1 is a hump-shaped function of the abundance of trophic level T, AND that, since $\left(1 - \frac{N_T}{K_T}\right) < 1$ (for N_T>0), the only way for N_{T+1}>N_T is if $\varphi \left(\frac{\overline{W}_T}{\overline{W}_{T+1}}\right)^{\beta} \frac{\delta_2 \delta_3}{\delta_1} > \left(1 - \frac{N_T}{K_T}\right)^{-1}$, and similarly for B_{T+1}>B_T: $\varphi \left(\frac{\overline{W}_{T+1}}{\overline{W}_T}\right)^{\beta-1} \frac{\delta_2 \delta_3}{\delta_1} > \left(1 - \frac{N_T}{K_T}\right)^{-1}$. Values of scaling constant δ_1 corresponding to the actual rate of energy expenditure by free-living organisms in nature (i.e. including all costs needed for individuals to contribute to maintaining a stable population) are yet inadequately

known, but the few reported values range from ≈200 J×day^{-1} for ectotherms (reptiles), via ≈5 000 J×day^{-1} for mammals to ≈10 000 J×day^{-1} for birds (based on field metabolic rates in Glazier 2010, converted from ml O_2×day^{-1} to J×day^{-1}). Moreover, values of the energy content of living tissue are variable, depending on the composition of the biomass, but typically range from 10^3 to 10^4 J×g^{-1} (with a standard value of 7×10^3 J×g^{-1} given by Peters 1983). Since δ_2 should represent whole organism energy concentration (i.e. averaged over the entire individual), characteristic values probably lie near the lower end of this range. This supposition is supported by data on sea ducks (Žydelis & Richman, 2015), for which the average whole organism energy concentration lie around 2×10^3 J×g^{-1}. However, by combining δ_1=[10^2 10^4] and δ_2=[10^3 10^4] with a reported value for δ_3 of approximately 0.2 day^{-1} (Fenchel 1974, Blueweiss et al. 1978) possible values for the ratio $\frac{\delta_2\delta_3}{\delta_1}$ are put in the range of 0.02 to 20. Furthermore, most prey-predator body mass ratios lie in the range from 10^{-5} to 10^1 (Brose et al. 2006). Based on this, Fig. I show the result of an analysis of the metabolic constraints on Eltonian pyramids, as formulated by Eqs. S1.10 and S1.11, within the potential ranges for the prey-predator body mass ratio and scaling constant ratio expected to occur in real systems. For numerical abundance, it is clear that for most combinations of the prey-predator body mass ratio and the scaling constant ratio, the requirements for the ingestion-assimilation efficiency to produce non-Eltonian patterns are unrealistic (Fig. IA, red area where φ need to be ≥1 for trophic level T+1 to have a higher abundance than trophic level T, for at least some level of abundance of trophic level T). If the prey-predator body mass ratio and/or the scaling constant ratio is large enough it could be possible for trophic level T+1 to have a higher numerical abundance than trophic level T, for some level of abundance of trophic level T, without the need for φ to be unrealistically high (blue to orange area in Fig. IA), but it is doubtful if these combinations of the prey-predator body mass ratio and scaling constant ratio occur in real systems. In fact, based on reported average prey-predator body mass ratios in different ecosystem types and for different consumer types (Brose et al. 2006), and estimates of plausible values for the scaling constant ratio, puts all these consumer-ecosystem type combinations in the orange-red area where φ needs to be unrealistically high for trophic level T+1 to have a higher numerical abundance than trophic level T (Fig. IA). Although acknowledging that these scaling constant ratios are coarse estimates (due to considerable uncertainty in the true values for δ_1 and δ_2 in particular), this serves to illustrate the difficulty to break a pyramidal pattern of numerical abundance. The abundance of trophic level T+1 show the same qualitative relationship with the abundance of trophic level T+1, irrespective of the prey-predator body mass ratio and scaling constant ratio, i.e. first increasing and peaking at an intermediate value end then decreasing, but for no consumer-ecosystem type combinations does the abundance of trophic level T+1 come anywhere near the abundance of trophic level T (Fig. IB).

For biomass abundance instead, the requirements for the ingestion-assimilation efficiency to produce non-Eltonian patterns are potentially realistic for many more combinations of the prey-predator body mass ratio and the scaling constant ratio (i.e. blue to orange area much larger in Fig. IC than in Fig. IA). Most consumer-ecosystem type combinations are found in

this realistic zone, which is characterized by a low prey-predator body mass ratio and/or high scaling constant ratio. The range of abundance of trophic level *T* for which $B_{T+1}>B_T$, however, highlights one additional requirement: trophic level *T*+1 must significantly reduce the abundance of level *T* since B_{T+1} will not be able to exceed B_T if the abundance of trophic level *T* is high enough. To summarize, these results reinforce the argument that Eltonian pyramids of numerical abundance should very much be the rule rather than the exception, while non-Eltonian pyramids of biomass abundance can be expected to be more frequent, also when consumers reduces the abundance of their resources.

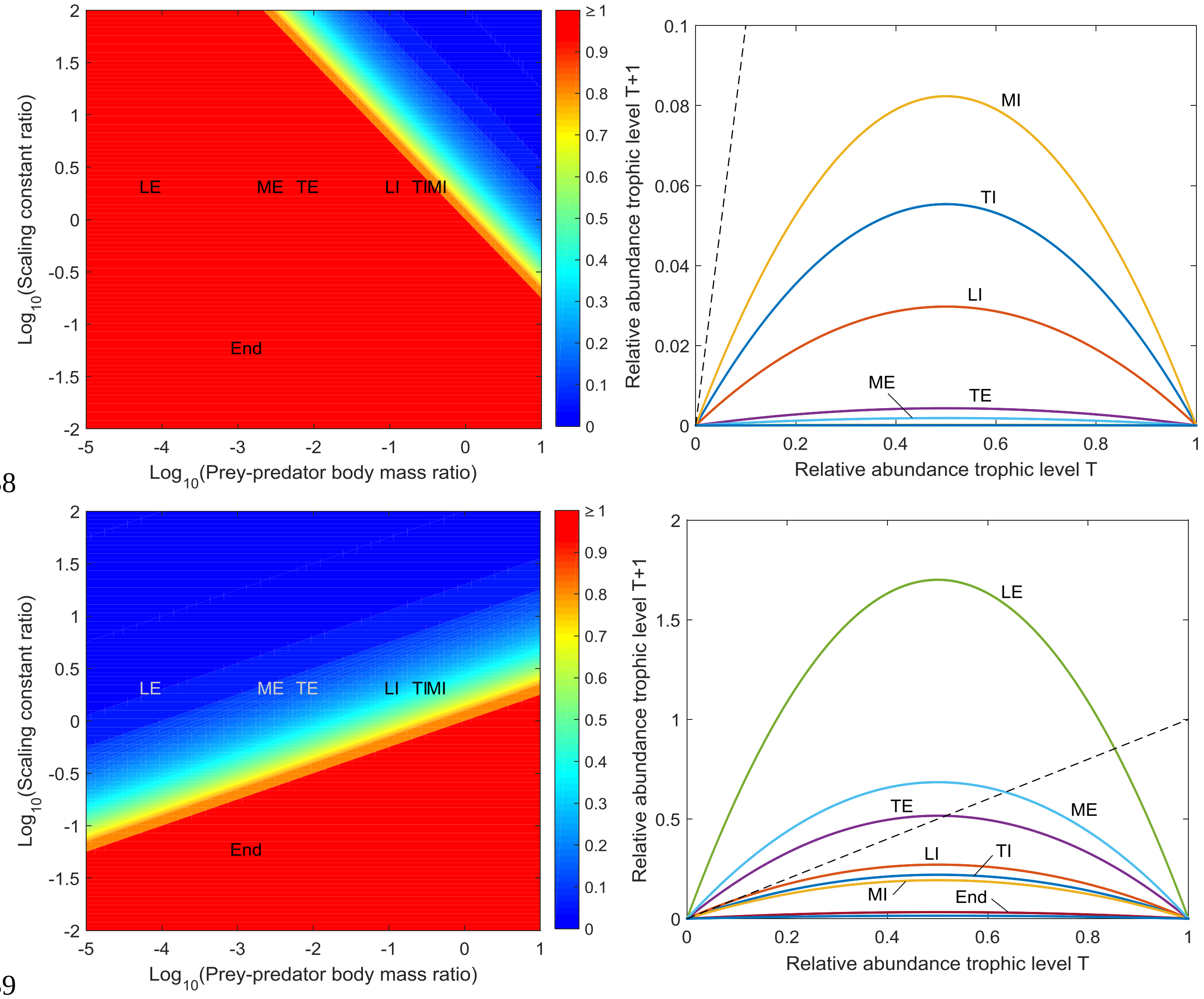

Fig. I Metabolic constraints on Eltonian pyramids. (*A*) & (*C*): Color show the minimum value of the ingestion-assimilation efficiency (φ) needed for trophic level *T*+1 to have a higher (*A*) numerical and (*C*) biomass abundance than trophic level *T*, for at least some level of abundance of trophic level *T*, for various combinations of the prey-predator body mass ratio ($\frac{\overline{W}_T}{\overline{W}_{T+1}}$) and scaling constant ratio ($\frac{\delta_2\delta_3}{\delta_1}$). For example, for combinations of the prey-predator body mass ratio and scaling constant ratio that lie in the red area, φ needs to be

greater than unity (which is biologically impossible) for trophic level T+1 to have a higher abundance than trophic level T, while in the green area it is sufficient that φ>0.5. Letter combinations give examples of the expected prey-predator body mass ratio and scaling constant ratio for three different consumer types (invertebrates, ectotherms and endotherms) in three types of ecosystems (lake, marine and terrestrial). (*B*) & (*D*): Examples of the relationship between the abundance on trophic level T+1 and trophic level T for the seven consumer and ecosystem types in (*A*) & (*C*). Dashed line represents the 1:1 line where the abundance on trophic level T+1 equals that on trophic level T. *X*-coordinates for each letter combination in (*A*) & (*C*) represent the average prey-predator body mass ratio for different consumer types in different ecosystems as reported in Brose *et al.* (2006). *Y*-coordinates were obtained by estimating the scaling constant ratio using the following values for δ_1, δ_2 and δ_3 (assuming no difference among ecosystem types): invertebrates: δ_1=192, δ_2=1000, δ_3=0.2; ectotherms: δ_1=192, δ_2=1000, δ_3=0.2, endotherms: δ_1=7000, δ_2=1000, δ_3=0.2. LI: lake invertebrates, MI: marine invertebrates, TI: terrestrial invertebrates, LE: lake ectotherms, ME: marine ectotherms, TE: terrestrial ectotherms, End: Endotherms.

Literature cited

Blueweiss et al. (1978) Relationships between body size and some life history parameters. *Oecologia* 37, 257-272.

Brose, U. *et al.* (2006) Consumer-resource body-size relationships in natural food webs. *Ecology,* 87, 2411-2417

Brown, J.H., Gillooly, J.F., Allen, A.P., Savage, V.M. & West, G.B. (2004) Towards a metabolic theory of ecology. *Ecology*, 85, 1771-1789.

Cohen, J.E., Pimm, S.L., Yodzis, P. and Saldaña, J. (1993) Body sizes of animal predators and animal prey in food webs. *J. Animal Ecol.*, 62, 67-78.

Fenchel, T. (1974) Intrinsic rate of natural increase: the relationship with body size. *Oecologia* 14, 317-326.

Glazier, D. S. (2010) A unifying explanation for diverse metabolic scaling in animals and plants. *Biological Reviews* 85, 111-138.

Gillooly, J.F., Brown, J.H., West, G.B., Savage, V.M. & Charnov, E.L. (2001) Effects of Size and Temperature on Metabolic Rate. *Science,* 293, 2248-2251.

Hemmingsen, A. M. (1950) The relation of standard (basal) energy metabolism to total fresh weight of living organisms. *Reports of the Steno Memorial Hospital and the Nordisk Insulinlaboratorium* 4, 1-48.

Kleiber, M. (1947) Body size and metabolic rate. *Physiological Reviews* 27,511-541.

Peters, R. H. (1983) *The ecological implications of body size*. Cambridge University Press, New York, NY, USA.

Teramoto, E. (1993) Dynamical structure of energy trophic levels. *Ecological Modelling,* 96, 135-147.

Žydelis R. & Richman S. (2015) Foraging behavior, ecology and energetics of sea ducks. in *Ecology and Conservation of North American Sea Ducks* (eds. Savard, J.-P. L., Derksen, D. V., Esler, D. & Eadie, J. M.). CRC Press, Boca Raton, TL, USA.

NOTE S2 - EQUILIBRIUM DENSITIES & PYRAMIDALITY

The equilibrium densities, N_i^*, as functions of the interaction coefficients, a_{ij}, and mortality rates, b_i, in two (Eq. S2.1), three (Eq. S2.2) and four level (Eq. S2.3) Lotka-Volterra food chains ($a_{ij} \geq 0$, $b_i \geq 0$, $i = 1 \ldots n$) are:

$$\text{(S2.1)} \quad \begin{cases} N_1^* = \dfrac{b_1 a_{22} + a_{12} b_2}{a_{22} a_{11} + a_{12} a_{21}} \\ N_2^* = \dfrac{a_{21} b_1 - b_2 a_{11}}{a_{22} a_{11} + a_{12} a_{21}} \end{cases}$$

$$\text{(S2.2)} \quad \begin{cases} N_1^* = \dfrac{b_1 a_{22} a_{33} + b_1 a_{23} a_{32} + b_2 a_{12} a_{33} - b_3 a_{12} a_{23}}{a_{11} a_{22} a_{33} + a_{11} a_{23} a_{32} + a_{21} a_{12} a_{33}} \\ N_2^* = \dfrac{b_1 a_{21} a_{33} - b_2 a_{11} a_{33} + b_3 a_{11} a_{23}}{a_{11} a_{22} a_{33} + a_{11} a_{23} a_{32} + a_{21} a_{12} a_{33}} \\ N_3^* = \dfrac{b_1 a_{21} a_{32} - b_2 a_{11} a_{32} - b_3 a_{11} a_{22} - b_3 a_{12} a_{21}}{a_{11} a_{22} a_{33} + a_{11} a_{23} a_{32} + a_{21} a_{12} a_{33}} \end{cases}$$

$$\text{(S2.3)} \quad \begin{cases} N_1^* = \dfrac{b_1 \left(a_{22} a_{33} a_{44} + a_{22} a_{34} a_{43} + a_{23} a_{32} a_{44}\right) + b_2 \left(a_{12} a_{33} a_{44} + a_{12} a_{34} a_{43}\right)}{a_{11} a_{22} a_{33} a_{44} + a_{11} a_{22} a_{34} a_{43} + a_{11} a_{23} a_{32} a_{44} + a_{21} a_{12} a_{33} a_{44} + a_{21} a_{12} a_{34} a_{43}} + \\ \quad + \dfrac{b_4 a_{12} a_{23} a_{34} - b_3 a_{12} a_{23} a_{44}}{a_{11} a_{22} a_{33} a_{44} + a_{11} a_{22} a_{34} a_{43} + a_{11} a_{23} a_{32} a_{44} + a_{21} a_{12} a_{33} a_{44} + a_{21} a_{12} a_{34} a_{43}} \\ N_2^* = \dfrac{b_1 \left(a_{21} a_{33} a_{44} + a_{21} a_{34} a_{43}\right) - b_2 \left(a_{11} a_{33} a_{44} + a_{11} a_{34} a_{43}\right)}{a_{11} a_{22} a_{33} a_{44} + a_{11} a_{22} a_{34} a_{43} + a_{11} a_{23} a_{32} a_{44} + a_{21} a_{12} a_{33} a_{44} + a_{21} a_{12} a_{34} a_{43}} \\ \quad + \dfrac{b_3 a_{11} a_{23} a_{44} - b_4 a_{11} a_{23} a_{34}}{a_{11} a_{22} a_{33} a_{44} + a_{11} a_{22} a_{34} a_{43} + a_{11} a_{23} a_{32} a_{44} + a_{21} a_{12} a_{33} a_{44} + a_{21} a_{12} a_{34} a_{43}} \\ N_3^* = \dfrac{b_1 a_{21} a_{32} a_{44} - b_2 a_{11} a_{32} a_{44} - b_3 \left(a_{11} a_{22} a_{44} + a_{12} a_{21} a_{44}\right)}{a_{11} a_{22} a_{33} a_{44} + a_{11} a_{22} a_{34} a_{43} + a_{11} a_{23} a_{32} a_{44} + a_{21} a_{12} a_{33} a_{44} + a_{21} a_{12} a_{34} a_{43}} \\ \quad + \dfrac{b_4 \left(a_{11} a_{22} a_{34} + a_{12} a_{21} a_{34}\right)}{a_{11} a_{22} a_{33} a_{44} + a_{11} a_{22} a_{34} a_{43} + a_{11} a_{23} a_{32} a_{44} + a_{21} a_{12} a_{33} a_{44} + a_{21} a_{12} a_{34} a_{43}} \\ N_4^* = \dfrac{b_1 a_{21} a_{32} a_{43} - b_2 a_{11} a_{32} a_{43} - b_3 \left(a_{11} a_{22} a_{43} + a_{12} a_{21} a_{43}\right)}{a_{11} a_{22} a_{33} a_{44} + a_{11} a_{22} a_{34} a_{43} + a_{11} a_{23} a_{32} a_{44} + a_{21} a_{12} a_{33} a_{44} + a_{21} a_{12} a_{34} a_{43}} + \\ \quad + \dfrac{- b_4 \left(a_{11} a_{22} a_{33} + a_{11} a_{23} a_{32} + a_{12} a_{21} a_{33}\right)}{a_{11} a_{22} a_{33} a_{44} + a_{11} a_{22} a_{34} a_{43} + a_{11} a_{23} a_{32} a_{44} + a_{21} a_{12} a_{33} a_{44} + a_{21} a_{12} a_{34} a_{43}} \end{cases}$$

Since the denominators in Eqs. (S2.1-S2.3) always are positive the conditions for feasibility is simply that the numerators in the same equations are positive. From this it can be seen that increasing b_1 increases the equilibrium densities at all trophic levels, with density dependent consumer mortality (DDCM) (i.e. if all $a_{ii} > 0$). But without DDCM, the equilibrium densities of odd-numbered species (i.e. species on an odd numbered trophic level) only, increase (with

increasing b_1) in odd-numbered food chains (i.e. food chains with an odd number of trophic levels), while even-numbered species increase in even-numbered food chains.

From the equilibrium densities the combined conditions for both feasibility (all $N_i^* > 0$) and pyramidality ($N_i^* > N_{i+1}^*$ for $i = 1$ to n-1) are easily derived. The condition for pyramidality is simply that the difference between the numerators in Eqs. (S2.1-S2.3) respectively, are positive and the *total* condition for both feasibility and pyramidality in Lotka-Volterra food chains of length two to four is thus:

$$\text{(S2.4)} \quad \begin{cases} b_1a_{22} + a_{12}b_2 = X_1^*(2) > b_1a_{21} - b_2a_{11} = X_2^*(2) > 0 \qquad (n = 2) \\ a_{33}X_1^*(2) + b_1a_{23}a_{32} - b_3a_{12}a_{23} > a_{33}X_2^*(2) + b_3a_{11}a_{23} > \\ \qquad a_{32}X_2^*(2) - b_3(a_{11}a_{22} + a_{12}a_{21}) > 0 \qquad (n = 3) \\ a_{44}X_1^*(3) + a_{34}a_{43}X_1^*(2) + b_4a_{12}a_{23}a_{34} > a_{44}X_2^*(3) + a_{34}a_{43}X_2^*(2) - b_4a_{11}a_{23}a_{34} > \\ \qquad a_{44}X_3^*(3) + b_4a_{34}(a_{11}a_{22} + a_{12}a_{21}) > a_{43}X_3^*(3) + \\ \qquad -b_4(a_{11}a_{22}a_{33} + a_{11}a_{23}a_{32} + a_{12}a_{21}a_{33}) > 0 \qquad (n = 4) \end{cases}$$

where $X_i^*(n)$ denotes the "equilibrium numerators" (i.e. $X_i^*(n) = \text{num}(N_i^*)$ for chain length n). From this it can be seen that the probability for pyramidality in Lotka-Volterra food chains of length two to four is higher with DDCM than without. More precisely, with DDCM in the top trophic level (i.e. if $a_{nn} > 0$), the restrictions on the interaction strengths and mortality rates that will ensure a food chain with n number of trophic levels to be both feasible and pyramidal is less severe if the "same" food chain, but of length n-1 (i.e. without the top trophic level), is pyramidal. For the most, this is also true with DDCM in other species (i.e. if $a_{ii} > 0$, $i = 2,\ldots n$-1), than without DDCM. For example, the conditions ensuring that $N_1^* > N_2^*$ and $N_2^* > N_3^*$ in a three level food chain is:

$$N_1^* > N_2^* \quad \Leftrightarrow \quad b_3a_{23}(a_{11} + a_{12}) - b_1a_{23}a_{32} < a_{33}\left(X_1^*(2) - X_2^*(2)\right) \qquad \text{(S2.5)}$$

$$N_2^* > N_3^* \quad \Leftrightarrow \quad X_2^*(2)(a_{32} - a_{33}) < b_3(a_{11}a_{23} + a_{11}a_{22} + a_{12}a_{21}) \qquad \text{(S2.6)}$$

Where $X_1^*(2) = b_1a_{22}+a_{12}b_2$ and $X_2^*(2) = b_1a_{21}-b_2a_{11}$ (i.e. $X_i^*(j) = \text{num}(N_i^*)$ for chain length j, or the "equilibrium numerator" for trophic level i in a food chain of length j). The conditions above are more easily fulfilled if a_{11}, a_{22} and a_{33} are positive and if the corresponding two level food chain is pyramidal (so that $X_1^*(2) > X_2^*(2)$). In an analogous way the probability for pyramidality in a four level chain is increased if the corresponding two and three level chains are pyramidal and both a_{33} and a_{44} are positive:

$$\begin{aligned} N_1^* > N_2^* \Leftrightarrow & -b_4a_{23}a_{34}(a_{12} + a_{11}) < \\ & a_{44}\left(X_1^*(3) - X_2^*(3)\right) + a_{34}a_{43}\left(X_1^*(2) - X_2^*(2)\right) \end{aligned} \qquad \text{(S2.7)}$$

$$\begin{aligned} N_2^* > N_3^* \Leftrightarrow & \, b_4a_{34}(a_{12}a_{21} + a_{11}a_{22} + a_{11}a_{23}) - a_{34}a_{43}X_2^*(2) < \\ & a_{44}\left(X_2^*(3) - X_3^*(3)\right) \end{aligned} \qquad \text{(S2.8)}$$

$$N_3^* > N_4^* \Leftrightarrow X_3^*(3)(a_{43} - a_{44}) < b_4 a_{11} a_{23} a_{32} + b_4 (a_{33} + a_{34})(a_{11} a_{22} + a_{12} a_{21}) \qquad \text{(S2.9)}$$

Here, the effect of a_{11} and a_{22} is somewhat ambiguous (as can be seen from Eq. S2.8). But, to summarize, pyramidality in shorter chains in combination with density dependent mortality at the top trophic level (at least) allow pyramidality for a larger range of parameter values (interaction strengths and mortality rates) than without DDCM.

NOTE S3 - CONSTANT MORTALITY RATES. NO DENSITY-DEPENDENT CONSUMER MORTALITY

Assuming

$$\begin{cases} a_{ii} = 0 (i = 2,...n) \\ b_i = b_2 \, (i = 3,...n) \\ b_1 = 1 \\ a_{11} = 1 \end{cases}, \qquad \text{(S3.1)}$$

the conditions for feasibility (Eqs. S3.2-S3.4) and pyramidality (Eqs. S3.5-S3.7) in two, three and four level Lotka-Volterra food chain can be simplified to:

$$\text{(S3.2)} \quad \begin{cases} N_1^* > 0 \Leftrightarrow b_2 > 0 \\ N_2^* > 0 \Leftrightarrow b_2 < a_{21} \end{cases} \quad (n = 2)$$

$$\text{(S3.3)} \quad \begin{cases} N_1^* > 0 \Leftrightarrow b_2 < \dfrac{a_{32}}{a_{12}} \\ N_2^* > 0 \Leftrightarrow b_2 > 0 \\ N_3^* > 0 \Leftrightarrow b_2 < \dfrac{a_{21}a_{32}}{a_{12}a_{21} + a_{32}} \end{cases} \quad (n = 3)$$

$$\text{(S3.4)} \quad \begin{cases} N_1^* > 0 \Leftrightarrow b_2 > 0 \\ N_2^* > 0 \Leftrightarrow b_2 < \dfrac{a_{21}a_{43}}{a_{23} + a_{43}} \\ N_3^* > 0 \Leftrightarrow b_2 > 0 \\ N_4^* > 0 \Leftrightarrow b_2 < \dfrac{a_{21}a_{32}a_{43}}{a_{23}a_{32} + a_{43}(a_{32} + a_{12}a_{21})} \end{cases} \quad (n = 4)$$

$$\text{(S3.5)} \quad N_1^* > N_2^* \Leftrightarrow b_2 > \frac{a_{21}}{1 + a_{12}} \quad (n = 2)$$

$$\text{(S3.6)} \quad \begin{cases} N_1^* > N_2^* \Leftrightarrow b_2 < \dfrac{a_{32}}{1 + a_{12}} \\ N_2^* > N_3^* \Leftrightarrow b_2 > \dfrac{a_{21}a_{32}}{a_{23} + a_{12}a_{21} + a_{32}} \end{cases} \quad (n = 3)$$

$$\text{(S3.7)} \quad \begin{cases} N_1^* > N_2^* \Leftrightarrow b_2 > \dfrac{a_{21}a_{43}}{a_{12}a_{23} + a_{43}(1 + a_{12}) + a_{23}} \\ N_2^* > N_3^* \Leftrightarrow b_2 < \dfrac{a_{21}a_{43}}{a_{23} + a_{12}a_{21} + a_{43}} \\ N_3^* > N_4^* \Leftrightarrow b_2 > \dfrac{a_{21}a_{32}a_{43}}{a_{12}a_{21}a_{34} + a_{23}a_{32} + a_{43}(a_{32} + a_{12}a_{21})} \end{cases} \quad (n = 4)$$

From these conditions it can be seen that odd-numbered species (i.e. species on an odd numbered trophic level) are always feasible (i.e. has a positive equilibrium density) in even-numbered food chains (i.e. food chains with an even number of trophic levels), while in odd-numbered food chains even-numbered species are always feasible (under the assumption in Eq. (S3.1) and provided that $b_2 > 0$). In addition, the conditions for *feasibility* in chains of different lengths do not seem to contradict each other to any larger extent. But looking at pyramidality and, for example, comparing (*i*) the condition ensuring that N_1^* is greater than N_2^* in a two level food chain (see Eq. S3.5) with the same condition in a three level chain (see Eq. S3.6), and (*ii*) the condition ensuring that N_2^* is greater than N_3^* in a three level chain (see Eq. S3.6) with the same condition in a four level chain (see Eq. S3.7), yields the following conditions respectively:

$$N_1^* > N_2^* \quad \Leftrightarrow \quad a_{21} < b_2\left(1 + a_{12}\right) < a_{32} \qquad \text{(S3.8)}$$

$$N_2^* > N_3^* \quad \Leftrightarrow \quad a_{32} < \frac{b_2\left(a_{23} + a_{12}a_{21}\right)}{a_{21} - b_2} < a_{43} \qquad \text{(S3.9)}$$

Finally, comparing the condition ensuring that N_1^* is greater than N_2^* in a two level food chain with the condition ensuring that N_2^* is greater than N_3^* in a four level food chain yields:

$$a_{21} + \frac{a_{23}}{a_{12}} < \frac{b_2\left(1 + a_{12}\right)\left(a_{23} + a_{43} + a_{12}a_{21}\right) - a_{21}a_{43}}{a_{12}a_{21}} < a_{43} \qquad \text{(S3.10)}$$

The implication of conditions in Eqs. (S3.8-S3.10) is that a necessary (but not sufficient) condition for simultaneous pyramidality in food chains with two, three and four trophic levels (under the assumption that $b_i = b_2$) is that

$$a_{21} < a_{32} < a_{43} \qquad \text{(S3.11)}$$

and the more similar in magnitude a_{21}, a_{32} and a_{43} are, the more difficult it is for conditions in Eqs. (S3.8-S3.10) to be fulfilled. Therefore, assuming

$$\begin{cases} a_{ii} = 0 \ (i = 2, \dots n) \\ b_i = b_2 \ \ (i = 3, \dots n) \\ a_{i+1,i} = a_{21} \ \ (i = 2, \dots n-1) \end{cases} \qquad \text{(S3.12)}$$

makes it obvious that the conditions for *pyramidality* in food chains of different lengths (Eqs. S3.5-S3.7) are not compatible. The condition for N_1^* to be greater than N_2^* in a two level food chain is now the exact opposite to the corresponding condition in a three level chain (Eq. S3.8), and similarly, the condition ensuring that N_2^* is greater than N_3^* in a three level food chain contradicts the same condition in a four level chain (Eq. S3.9). Furthermore, for the conditions ensuring that N_1^* is greater than N_2^* in a two level food chain and N_2^* greater than N_3^* in a four level food chain (Eq. S3.10), both to be fulfilled requires that $a_{23} < 0$ which violates the basal assumptions of the model (Eq. 1). Thus, under the assumptions that $a_{i+1,i} = a_{21}$ and $b_i = b_2$ ($i = 2,\dots n$), it is not possible for LV food chains of adjacent lengths to be pyramidal, without density dependent consumer mortality (DDCM) (i.e. if $a_{ii} = 0$,

$i = 2,\ldots n$). These assumptions mean that, as an initial simplification, the resource interaction strengths within a food chain all are of the same magnitude and, thus, can be considered to be constant. The same applies to the consumer mortality rates. These possibly unrealistic assumptions are relaxed in Note S4. To summarize, without DDCM and assuming constant mortality rates and resource interaction strengths, chains of length two and three, or three and four, cannot be pyramidal simultaneously, for any range of the parameters b_1, b_2, a_{11}, a_{12}, a_{23} and a_{34}. But even if the resource interaction strengths are not assumed to be identical in magnitude, it is difficult for the conditions ensuring feasibility and pyramidality to be fulfilled in chains of adjacent lengths simultaneously, as conditions in Eqs. (S3.8-S3.10) imply. With DDCM ($a_{ii} > 0$, for all i) however, the conditions for pyramidality are more easily fulfilled and it is possible for food chains of different lengths to be pyramidal simultaneously.

NOTE S4 - CHANGING INTERACTION STRENGTHS & MORTALITY RATES

Assuming

$$\begin{cases} a_{i,i+1} = k_{pred}^{i-1} a_{1,2} \ (i = 1,...n-1) \\ a_{i+1,i} = k_{prey}^{i-1} a_{2,1} \ (i = 1,...n-1) \\ b_i = k_b^{i-2} b_2 \qquad (i = 2,...n-1) \\ b_1 = 1 \\ a_{11} = 1 \end{cases}, \tag{S4.1}$$

the general condition for pyramidality in linear Lotka-Volterra food chains is that $\frac{h}{|h|} b_2 > \frac{h}{|h|} \cdot \frac{g}{h} = \frac{g}{|h|}$ (with the additional requirement that $b_2 > 0$), where g and h are functions of the mortality rates and interaction strengths. In Lotka-Volterra chains of length two, three and four, the functions g and h are:

$$\text{(S4.2)} \quad N_1^* > N_2^* \Leftrightarrow \begin{cases} g = a_{21} - a_{22} \\ h = 1 + a_{12} \end{cases} \qquad (n = 2)$$

$$\text{(S4.3)} \quad \begin{cases} N_1^* > N_2^* \Leftrightarrow \begin{cases} g = a_{33}(a_{21} - a_{22}) - a_{12}a_{21}k_{prey}k_{pred} \\ h = (1 + a_{12})(a_{33} - k_{pred}a_{12}k_b) \end{cases} \\ N_2^* > N_3^* \Leftrightarrow \begin{cases} g = a_{21}(k_{prey}a_{21} - a_{33}) \\ h = a_{12}k_{pred}k_b + a_{12}a_{21}k_b + a_{21}k_{prey} + a_{22}k_b - a_{33} \end{cases} \end{cases} \qquad (n = 3)$$

$$\text{(S4.4)} \quad \begin{cases} N_1^* > N_2^* \Leftrightarrow \begin{cases} g = a_{12}a_{21}k_{prey}^2 k_{pred}^2 (a_{21} - a_{22}) + \\ \qquad -a_{44}(a_{22}a_{33} + a_{12}a_{21}k_{prey}k_{pred} - a_{33}a_{21}) \\ h = a_{12}a_{21}k_{pred}^2 k_{prey}^2 (a_{12} + 1) + a_{12}^2 k_{pred}^3 k_b^2 + \\ \qquad + a_{44}(a_{12} + 1)(a_{33} - a_{12}k_b k_{pred}) \end{cases} \\ N_2^* > N_3^* \Leftrightarrow \begin{cases} g = a_{44}(a_{21}^2 k_{prey} - a_{21}a_{33}) - a_{21}^2 a_{12} k_{prey}^2 k_{pred}^2 \\ h = a_{44}(k_b k_{pred} a_{12} - a_{33} + a_{21}k_{prey} + a_{22}k_b + a_{12}a_{21}k_b) + \\ \qquad -a_{22}k_{pred}^2 k_b^2 a_{12} - a_{12}a_{21}k_{pred}^2 k_{prey}^2 + \\ \qquad -a_{12}^2 k_{pred}^2 k_{pred} k_b^2 - a_{12}^2 a_{21} k_{pred}^2 k_b^2 \end{cases} \\ N_3^* > N_4^* \Leftrightarrow \begin{cases} g = a_{21}^2 k_{prey}(k_{prey}^2 a_{21} - a_{44}) \\ h = k_b^2 a_{12}a_{21}k_{pred}(a_{12}k_{pred} + k_{prey}) + a_{21}^2 k_{prey}^2 (k_{prey} + a_{12}k_b) + \\ \qquad -a_{44}k_{prey}a_{21} + (a_{33}k_b^2 - a_{44}k_b)(a_{22} + a_{12}a_{21}) + \\ \qquad + a_{22}k_b(a_{12}k_{pred}^2 k_b + a_{21}k_{prey}^2) \end{cases} \end{cases} \qquad (n = 4)$$

This means that unless $\begin{cases} b_2 > \dfrac{g}{h} \text{ if } h > 0 \\ \quad or \\ b_2 < \dfrac{g}{h} \text{ if } h < 0 \end{cases}$, N_i^* will not be greater than N_{i+1}^* (which is required for the food chain to be pyramidal). For example, for N_1^* to be greater than N_2^* in a three level chain, it is required that:

$$\text{(S4.5)} \quad \begin{cases} b_2 > \dfrac{a_{33}(a_{21}-a_{22})-a_{12}a_{21}k_{res}k_{cons}}{(1+a_{12})(a_{33}-k_{cons}a_{12}k_b)} & if \quad (a_{33}-k_{cons}a_{12}k_b) > 0 \\ b_2 < \dfrac{a_{33}(a_{21}-a_{22})-a_{12}a_{21}k_{res}k_{cons}}{(1+a_{12})(a_{33}-k_{cons}a_{12}k_b)} & if \quad (a_{33}-k_{cons}a_{12}k_b) < 0 \end{cases}.$$

This means that unless $k_{cons} = 1$, $k_{\mathrm{res}} = 1$ and $k_b = 1$, there are no general contradictions between the conditions for pyramidality in chains of different lengths, neither with, nor without density dependent consumer mortality (DDCM). Instead, it is obvious that whether a particular chain length is pyramidal or not depends in a complicated way on an interaction between the parameters b_2, k_{cons}, k_{res}, k_b, a_{12} and a_{ii}. However, assuming no DDCM (i.e. $a_{ii} = 0$, $i > 1$) the conditions for pyramidality in two, three and four level LV food chains above (i.e. Eqs. S4.2-S4.4) can be simplified to:

$$\text{(S4.6)} \quad N_1^* > N_2^* \Leftrightarrow b_2 > \frac{a_{21}}{1+a_{12}}$$

$$\text{(S4.7)} \quad \begin{cases} N_1^* > N_2^* \Leftrightarrow b_2 < \dfrac{a_{21}k_{res}}{(1+a_{12})k_b} \\ N_2^* > N_3^* \Leftrightarrow b_2 > \dfrac{a_{21}^2 k_{res}}{a_{12}k_{cons}k_b + a_{12}a_{21}k_b + a_{21}k_{res}} \end{cases}$$

$$\text{(S4.8)} \quad \begin{cases} N_1^* > N_2^* \Leftrightarrow b_2 > \dfrac{a_{21}^2 k_{res}^2}{(1+a_{12})(a_{21}k_{res}^2 + a_{12}k_{cons}k_b^2)} \\ N_2^* > N_3^* \Leftrightarrow b_2 < \dfrac{a_{21}^2 k_{res}^2}{a_{12}k_{cons}k_b^2 + a_{12}a_{21}k_b^2 + a_{21}k_{res}^2} \\ N_3^* > N_4^* \Leftrightarrow b_2 > \dfrac{a_{21}^3 k_{res}^3}{k_b^2 a_{12}a_{21}k_{cons}(a_{12}k_{cons}+k_{res}) + a_{21}^2 k_{res}^2 (k_{res}+a_{12}k_b)} \end{cases}$$

By reanalyzing the conditions that were incompatible under assumptions in Eq. (S3.12), it turns out that the criteria allowing food chains of adjacent lengths, without DDCM, to be pyramidal simultaneously, is

$$\frac{k_{res}}{k_b} > 1 \qquad \text{(Eq. S4.9)}$$

That is, both $k_{res} > 1$ and $k_b < 1$ promotes the existence of pyramidality in Lotka-Volterra food chains by creating an interval for b_2 for which the otherwise incompatible conditions are compatible, the larger the ratio k_{res}/k_b the larger the possible interval for b_2 (or in other words, the less restrictions on the value of b_2). Allowing the consumer per capita effects to decrease with increasing trophic height on the other hand does not affect the conditions that are contradictory with $k_b = 1$ and $k_{res} = 1$. To summarize, the overall effect of lowering the mortality rates of higher level consumers, at the same time as resource interaction strengths increase with increasing trophic height, is to increase the pyramidal overlap between chains of adjacent lengths.

Note S5 – Randomly drawn Interaction Strengths & Mortality Rates

The analytical results have been complemented by simulations where the mortality rates and interaction strengths have been drawn at random. The motivation for this was to see if the criteria for pyramidality, or 'deletion robust pyramidality' (see below), would create food chains with distributions of mortality rates or interaction strengths that are biased compared to random distributions (e.g. in the direction suggested by Eq. S4.9). Thus, a large number (n=1000) of model food chains were constructed by selecting the individual interaction strengths and mortality rates randomly from the following intervals:

$$\begin{cases} a_{i,i+1} = \begin{bmatrix}100 & 0\end{bmatrix} \\ a_{i+1,i} = \begin{bmatrix}0 & 1\end{bmatrix} \end{cases} \tag{S5.1}$$

$$\log|b_i| = \left[-2.5 - 0.5^{i-2} \quad -1.5 - 0.5^{i-2}\right]. \tag{S5.2}$$

This set the mean ratio between the consumer and resource interaction strengths to 100 in the original, unbiased data set from which feasible, pyramidal and species-deletion pyramidal chains were sampled.

The number of feasible chains (i.e. chains with a positive equilibrium for every trophic level) as well as the number of chains that were both feasible and pyramidal was then determined. (In linear food chain models of the LV type a feasible equilibrium is always globally stable Harrison 1979). The next step was to delete the top trophic level in every chain that was both feasible and pyramidal and determine how many of these chains that were feasible and pyramidal, still after this operation. These chains possess the properties of 'deletion robust feasibility' and 'deletion robust pyramidality' respectively.

To start with, there is little difference in the fraction of feasible chains in LV food chain models with or without density-dependent consumer mortality (Table S1). For this particular parameter setting the fraction of feasible chains is slightly higher for model chains without density-dependent mortality. The fraction of feasible chains, however, decreases quickly as chain length increases in both models (which is well in line with the early finding of Pimm and Lawton (1977) that the probability of feasibility decreases with increasing food chain length). The fraction of chains that are both pyramidal and feasible at first increases slightly and then decreases with increasing chain length, both with and without density-dependent consumer mortality, but the fraction of pyramidal chains is far greater if density-dependent mortality is incorporated in the consumers (Table S1). Interestingly, every pyramidal chain is 'species-deletion feasible' in the sense that it can endure a loss of the top species and still be feasible. Still, even more important, the probability of finding a LV food chain that is pyramidal both before and after a deletion of the top species (i.e. 'species-deletion pyramidal') is significantly higher with density-dependent consumer mortality. The fraction of all chains that are species-deletion pyramidal is never greater than 5% for chains without density-dependent mortality but lies between 15% and 40% in chains with density-dependent mortality (Table S1) except for the longest chain (n = 9) where the fraction of feasible chains (which sets the upper bound to the fraction of species-deletion pyramidal chains) have decreased to 11%. The pattern is even clearer when looking at the fraction of the *feasible*

chains that are species-deletion pyramidal (Fig. S9*A*) or the fraction of the *pyramidal* chains that are species-deletion pyramidal (Fig. S9*B*). When trophic interaction strengths are drawn at random from prescribed intervals it is far more likely that the food chain will be both feasible and pyramidal as well as capable of losing the top species and still be both feasible and pyramidal (i.e. species-deletion pyramidal) if the consumers show density-dependent mortality. Thus, the results in the previous sections are confirmed also when mortality rates and interaction strengths have been drawn at random.

An analysis of the relationship between the mean consumer and resource interaction strengths and food chain length in those chains that are feasible, pyramidal and species deletion pyramidal reveals an interesting pattern. Mean consumer interaction strength (mean$|a_{i,i+1}|$) is significantly negatively correlated and mean resource interaction strength (mean($a_{i+1,i}$)) positively correlated to the length of the food chains (Table S2; Fig. S10). Mean resource interaction strength increases from a smaller value than expected ($E(a_{i+1,i}) = 0.5$) in short chains, to slightly larger than the expected value in the longest chains, while mean consumer interaction strength (i.e. mean$|a_{i,i+1}|$) decreases from the expected value ($E(|a_{i,i+1}|) = 50$) to a slightly smaller value than expected in the longest chains. The effect is small for feasible chains, more pronounced for pyramidal chains and greatest for species-deletion pyramidal chains (Fig. S10). Thus, in order for model food chains to be pyramidal and species-deletion pyramidal, (it seems that) resource interaction strengths need to be smaller than expected in short chains while consumer interaction strengths need to be smaller and resource interaction strengths larger than expected in long food chains. This means that the average interaction strength ratio (i.e., $q = \frac{mean|a_{i,i+1}|}{mean(a_{i+1,i})}$) decreases with increasing chain length from larger than expected ($E(q) = 100$) in short chains to smaller than expected in long chains.

It has previously been shown (Jonsson and Ebenman, 1998) that if the interaction strength ratio decreases with increasing *trophic position* within a food chain (by increasing resource and decreasing consumer interaction strengths with increasing trophic position), the result is shorter return times to equilibrium of those chains, when compared to chains with a constant interaction strength ratio. However, it does not seem that the decreasing mean interaction strength ratio with increasing food chain length in the model chains reported here, result from decreasing consumer and increasing resource interaction strengths with increasing trophic position, within these chains. Fig. S11 show the mean consumer and resource interaction strengths at different trophic positions (within the chain) for different chain lengths and for those model chains that are species-deletion pyramidal. The statistical analysis (Table S3) does not, reveal any significant trends for the consumer or resource interaction strengths with respect to trophic position.

To summarize, when trophic interaction strengths and mortality rates are drawn at random from prescribed intervals it is far more likely that the food chain will be both feasible and pyramidal, as well as capable of losing the top species and still be both feasible and pyramidal (i.e. species-deletion pyramidal), with density dependent consumer mortality (DDCM) than

without. Furthermore, mean resource interaction strength and consumer mortality rates are significantly positively and negatively correlated respectively, to the trophic position in model chains, both with and without DDCM. The effect is most pronounced, in species-deletion pyramidal chains without DDCM. The correlations between mean consumer interaction strength and trophic position are all non-significant Increasing resource interaction strengths and decreasing consumer mortality rates, with increasing trophic position, both seem to increase the probability that a food chain is feasible, pyramidal as well as species-deletion pyramidal, especially without DDCM. Thus, the generality of the analytical results in Appendices S2-S4 are confirmed also for the situation where interaction strengths and mortality rates are allowed to vary randomly within a food chain.

Literature cited

Jonsson T. & Ebenman B (1998) Effects of predator-prey body size ratios on the stability of food chains. *Journal of Theoretical Biology* 193:407-417.

Harrison G. W. (1979) Global stability of food chains. *American Naturalist* 114:455-457.

Pimm S. L. & Lawton J. H. (1977) Number of trophic levels in ecological communities. *Nature* 268:329-331.

FIGURE S1

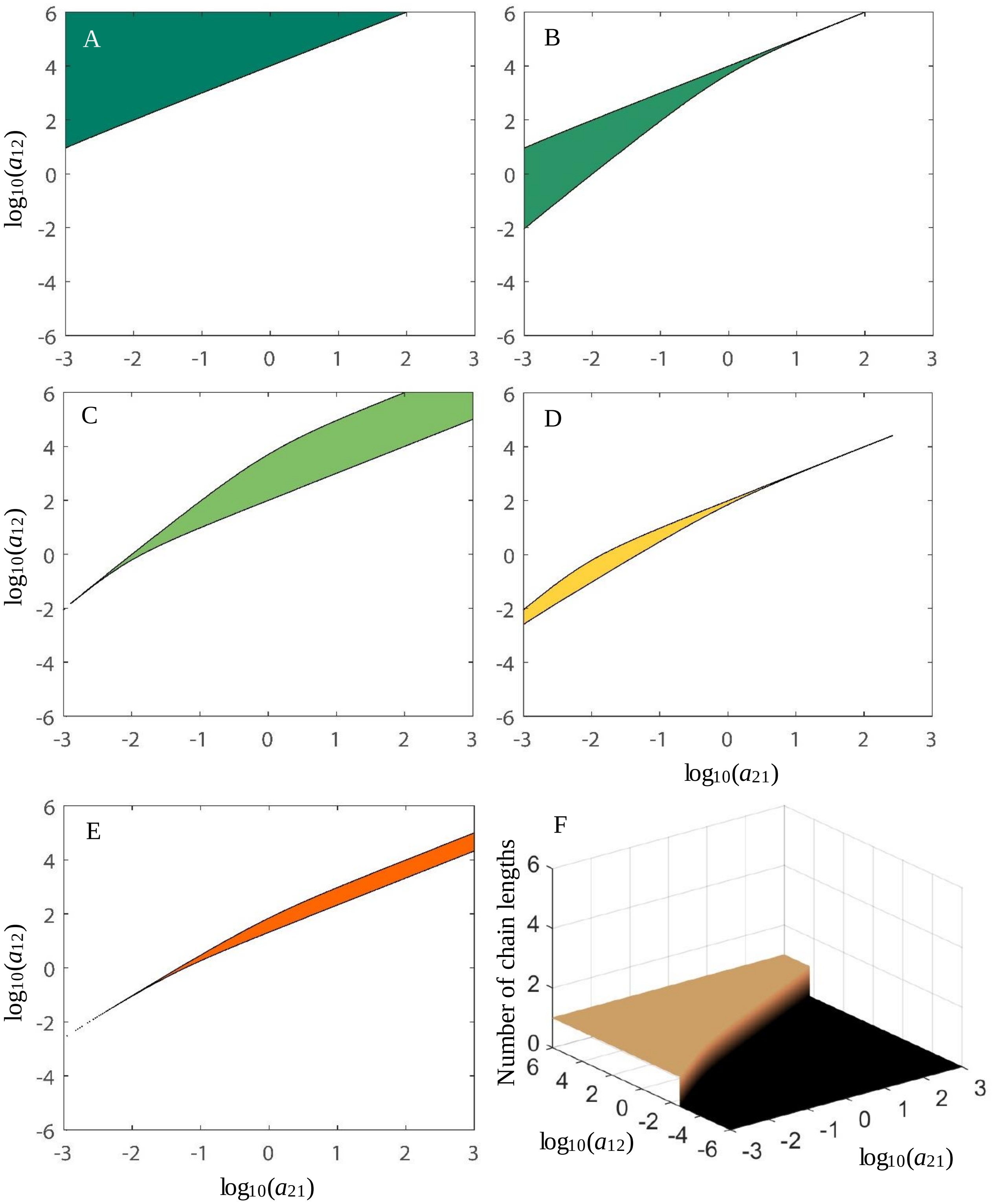

FIG. S1. The presence of Eltonian pyramids in Lotka-Volterra food chains with different number of trophic levels, without density-dependent consumer mortality. (*A*)-(*E*): Filled areas show the combinations of resource (a_{21}) and consumer (a_{12}) interaction strengths allowing for pyramidality in Lotka-Volterra food chains with two (*A*), three (*B*), four (*C*), five (*D*) and six (*E*) trophic levels, when resource and consumer interaction strengths are assumed to be independent of each other. (*F*): The number of food chain lengths (as indicated on the *z*-axis),

from two to six trophic levels, that are pyramidal simultaneously, for each combination of resource (a_{21}) and consumer (a_{12}) interaction strengths. Because the parameter regions allowing for pyramidality, in food chains with two to six trophic levels (subplots *A*-*E*), in this case do not overlap, the values on the *z*-axis only range between zero (= no food chain length is pyramidal) and unity (= only one food chain length is pyramidal). For example, in the lower right corner of subplot *F* (low values of both a_{21} and a_{12}) no food chain is pyramidal, while in the upper left as well as right corners only one food chain length is pyramidal (in this case a two trophic level chain for low values of a_{21} and high values of a_{12}, see subplot *A*, and a four trophic level chain for high values of both a_{21} and a_{12}, see subplot *C*). Fill colors in subplots *A*-*E* correspond to color scheme used for unhatched areas in Figs 1*A*-*B* and 2*A*-*B* (i.e. parameter regions where only one particular food chain length is pyramidal). Parameter settings: $a_{11} = 1$, $a_{ii} = 0$ ($i \geq 2$), $b_1 = 1$, $b_i = 0.0001$ ($i \geq 2$), $a_{i,i+1} = a_{12}$ and $a_{i+1,i} = a_{21}$.

FIGURE S2

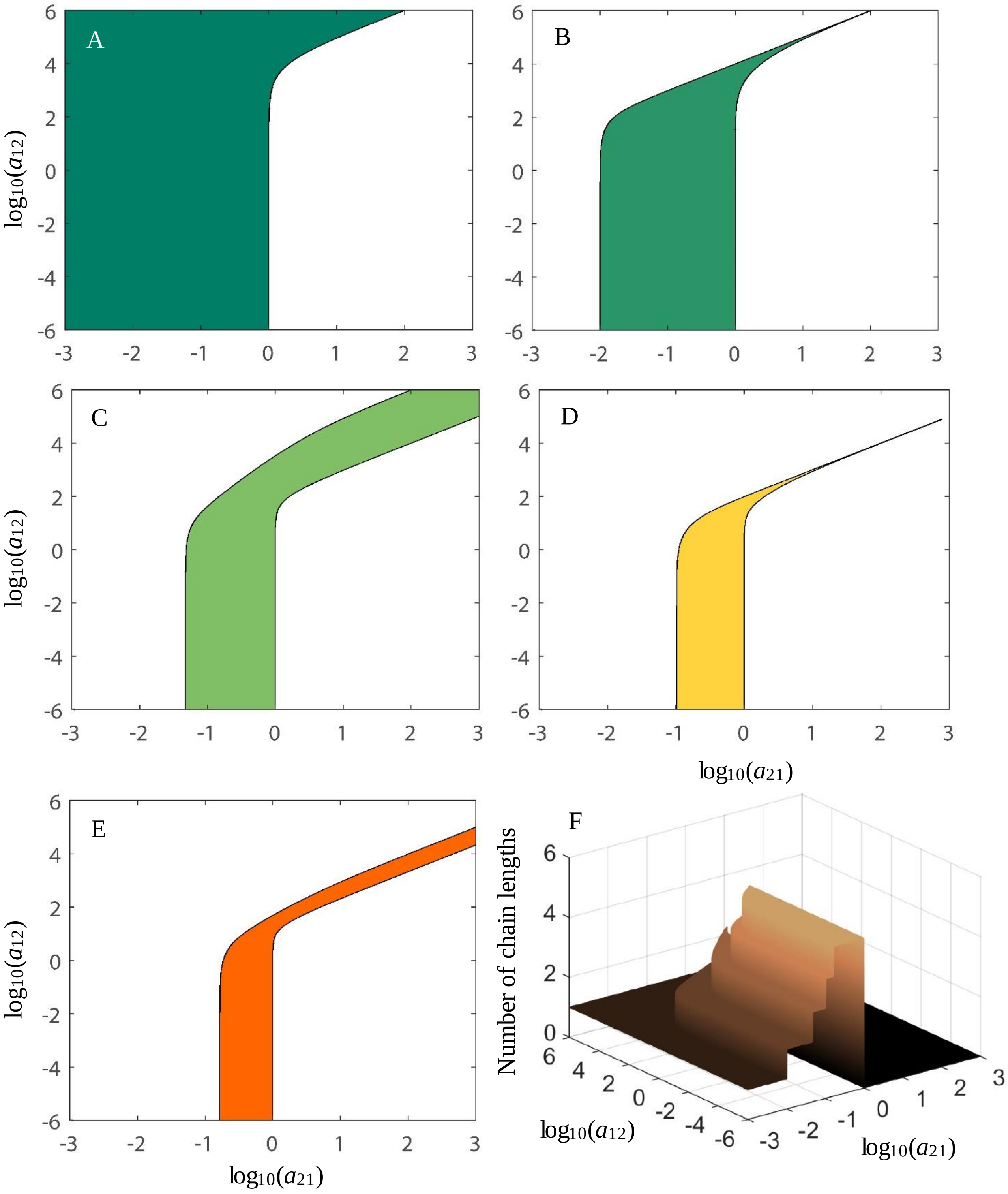

FIG. S2. Same as Fig. S1 except for $a_{ii} = 1$ ($i \geq 2$), i.e. Lotka-Volterra food chains with density-dependent consumer mortality. Because there is considerable overlap in the parameter regions allowing for pyramidality (subplots *A*-*E*) for intermediate values of a_{21} and a_{12}, the values on the *z*-axis of subplot *F* in this case range between zero (= no food chain length is pyramidal) and five (= all five food chain lengths between two to six trophic levels are pyramidal).

FIGURE S3

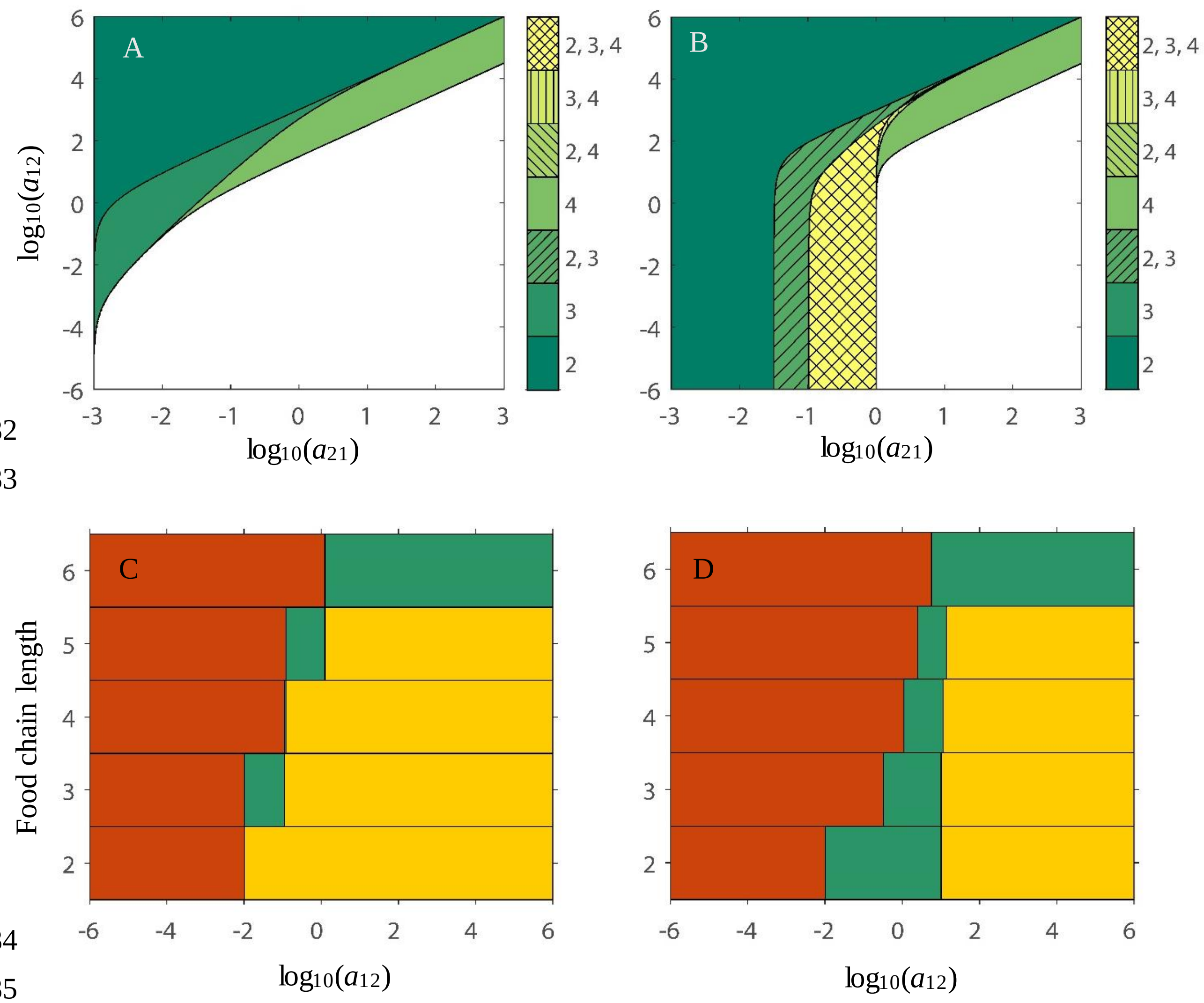

FIG. S3. Same as Fig. 1 except for $b_i = 0.001$ $(i \geq 2)$.

FIGURE S4

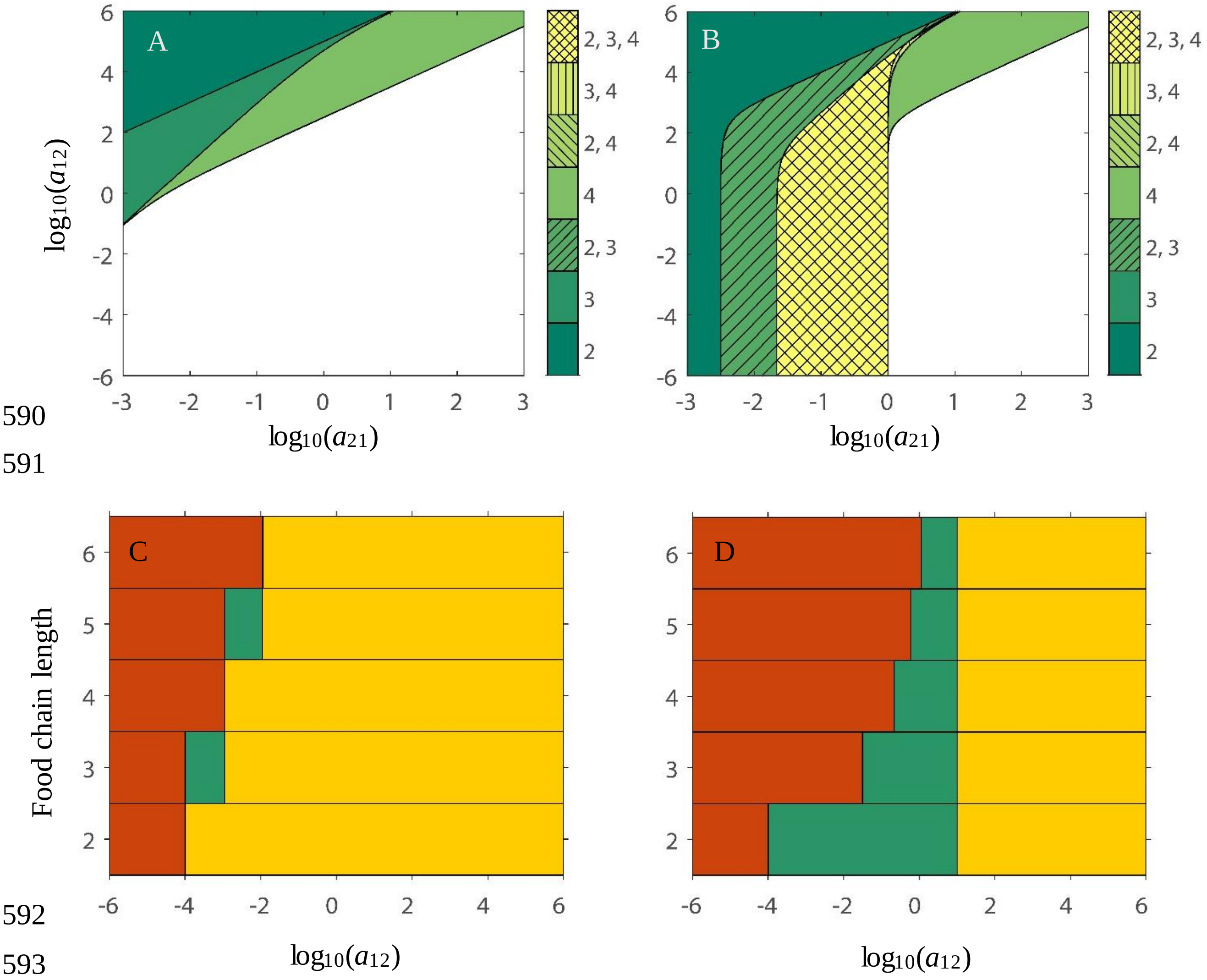

FIG. S4. Same as Fig. 1 except for $b_i = 0.00001$ $(i \geq 2)$.

FIGURE S5

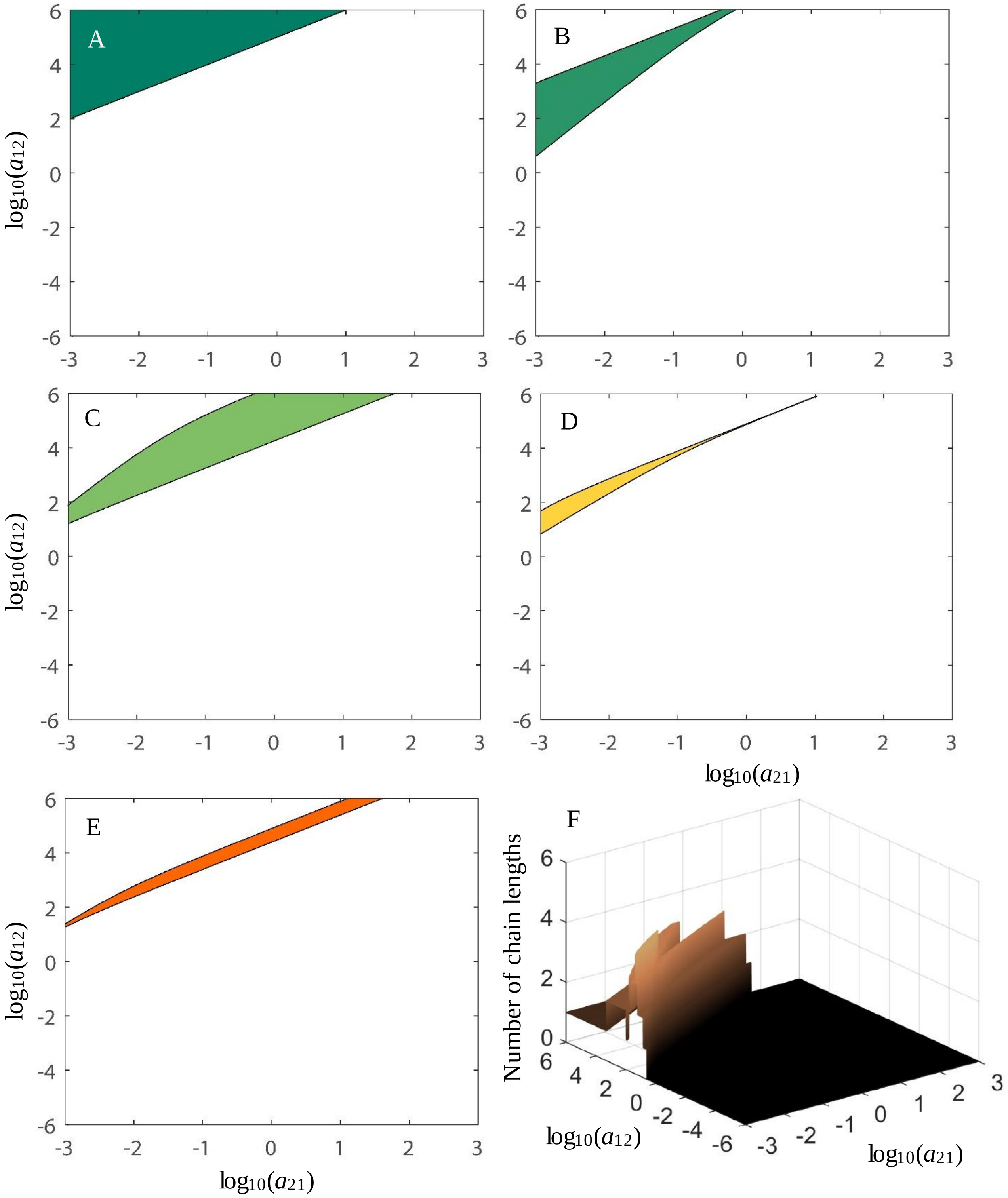

FIG. S5. Same as Fig. S1 except for $a_{i+1,i} = k_{res}^{i-1} a_{2,1}$, $a_{i,i+1} = k_{cons}^{i-1} a_{1,2}$ and $b_i = k_b^{i-2} b_2$ $(i \geq 2)$, where $k_{res} = 2$, $k_{cons} = 0.5$ and $k_b = 0.1$. That is, resource interaction strengths ($a_{i+1,i}$) and consumer mortality rates (b_i) decrease and consumer interaction strengths ($a_{i,i+1}$) increase with trophic height. Because (compared to Fig. S1) there now is some overlap in the parameter regions allowing for pyramidality (subplots *A*-*E*) for relatively low and high values,

respectively, of a_{21} and a_{12}, the values on the z-axis of subplot F in this case range between zero (= no food chain length is pyramidal) and four (= four out of five food chain lengths between two to six trophic levels are pyramidal).

FIGURE S6

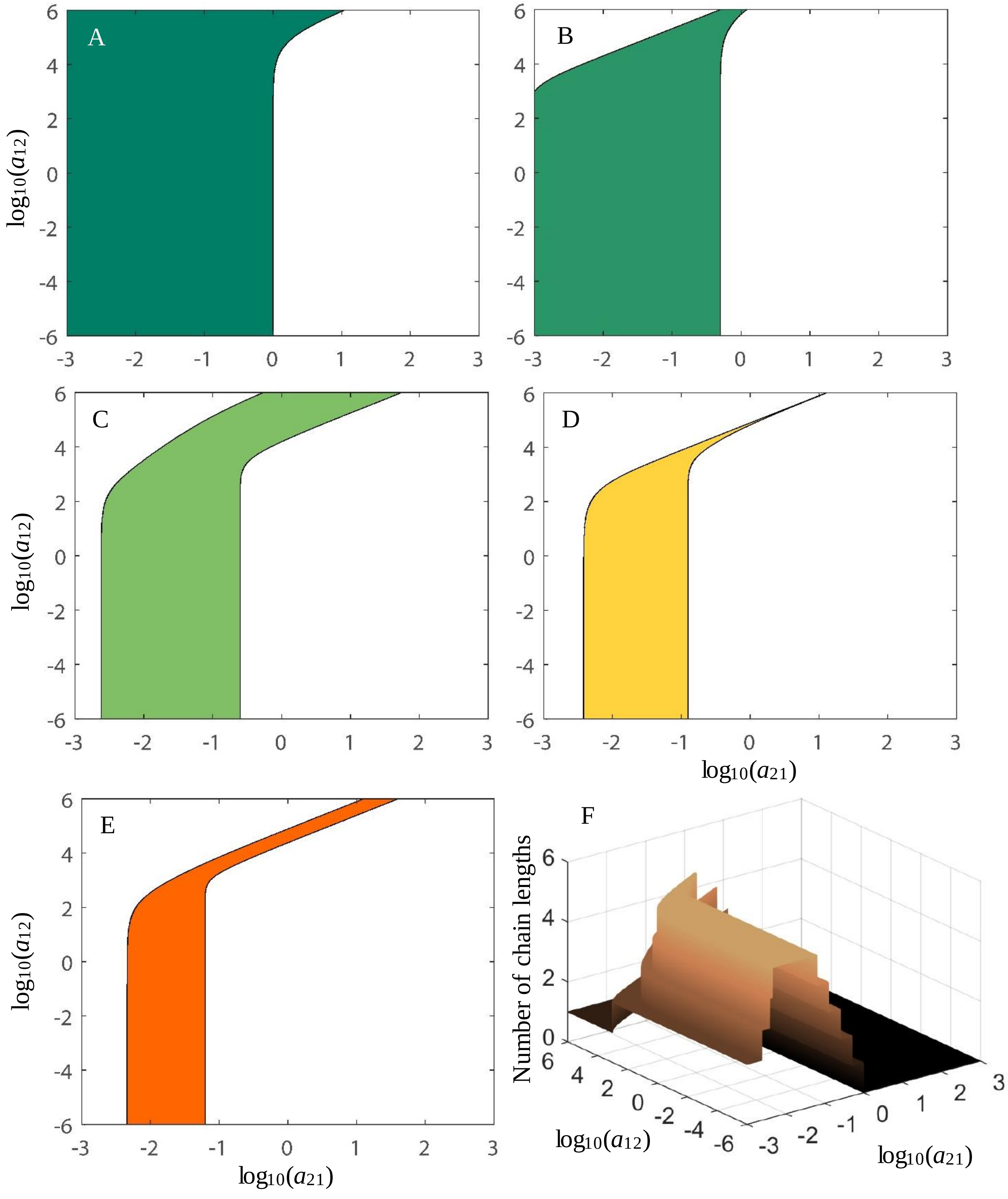

FIG. S6. Same as Fig. S5 except for $a_{ii} = 1$ ($i \geq 2$), i.e. Lotka-Volterra food chains with density-dependent consumer mortality.

FIGURE S7

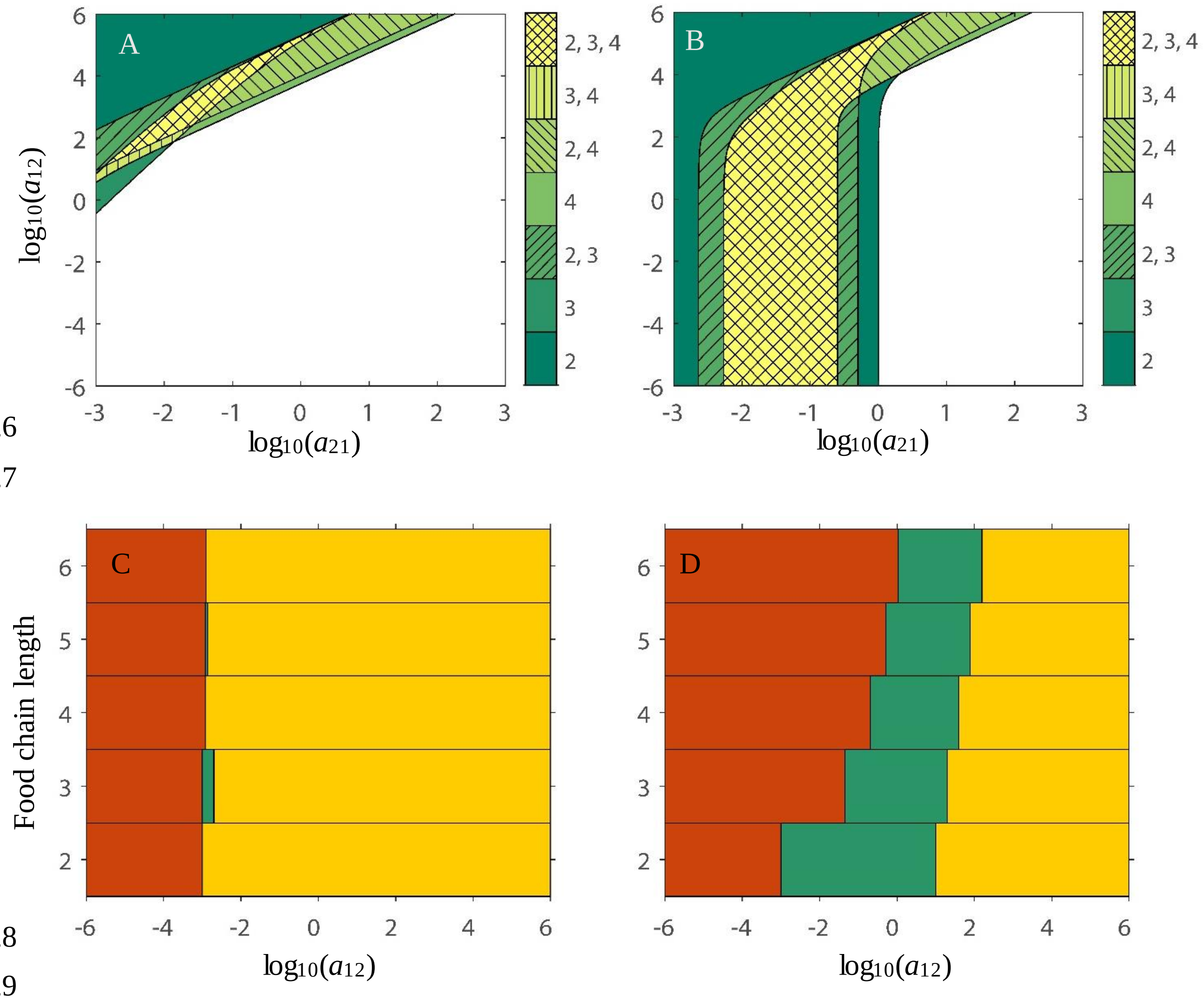

FIG. S7. Same as Fig. 2 except for $b_i = 0.001$ $(i \ge 2)$.

FIGURE S8

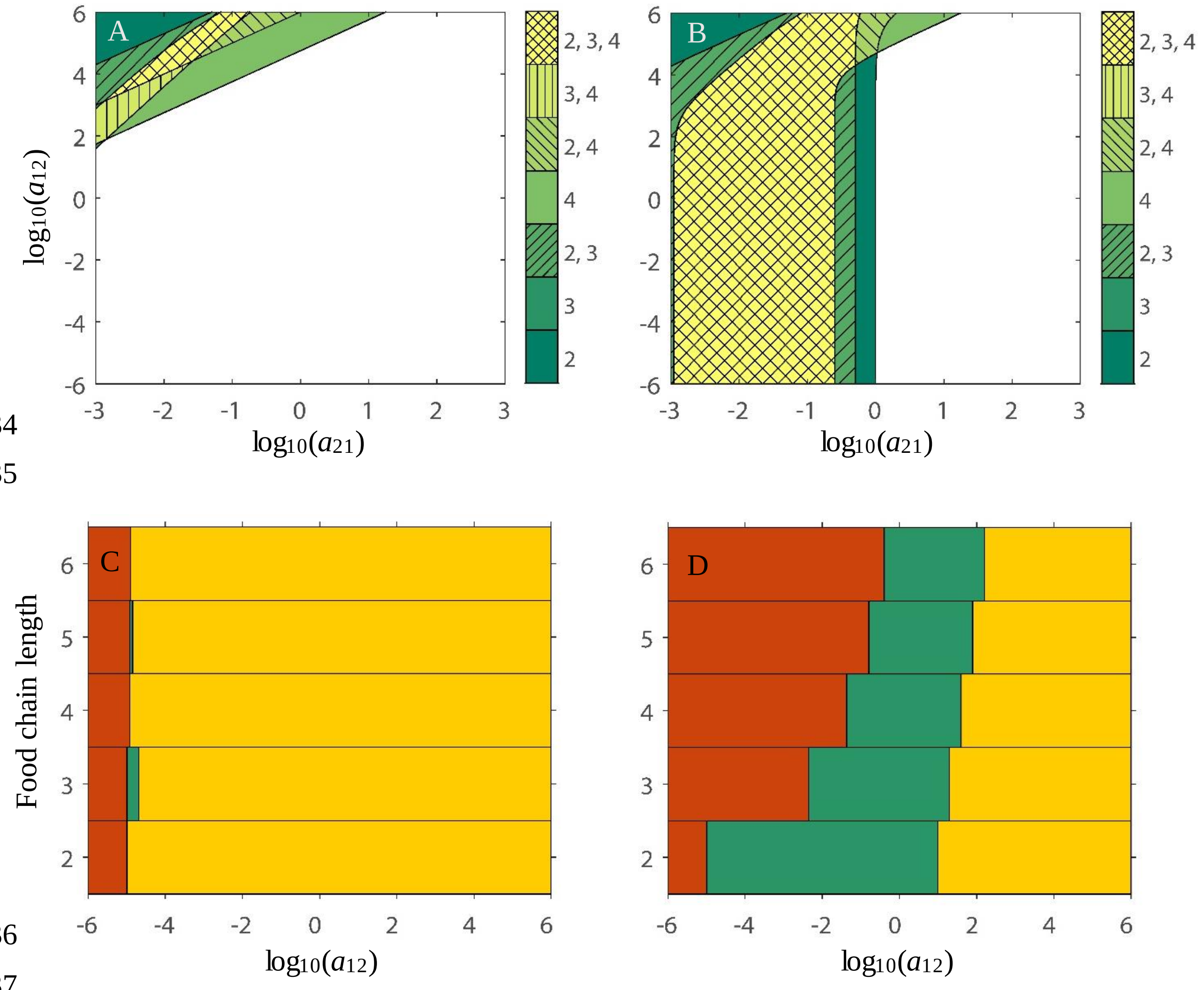

FIG. S8. Same as Fig. 2 except for $b_i = 0.00001$ $(i \geq 2)$.

FIGURE S9

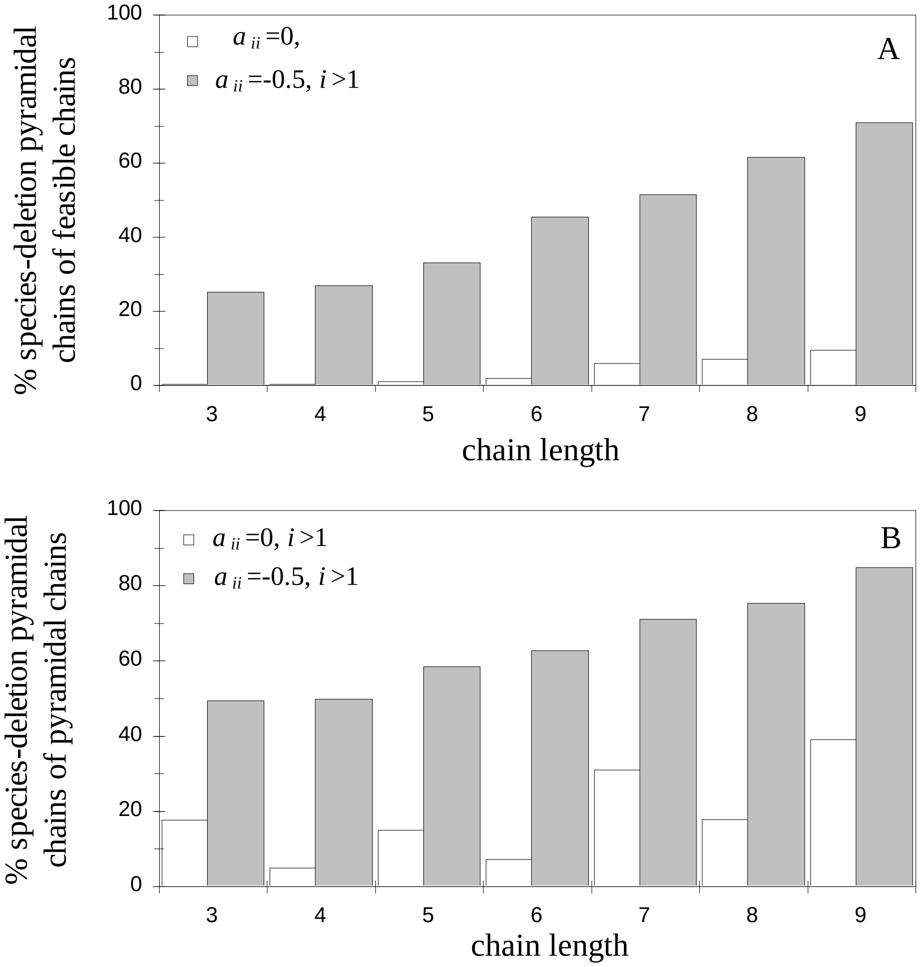

Fig. S9 (A) The fraction of the feasible chains, for Lotka-Volterra food chains with 3 to 9 trophic levels that are species-deletion pyramidal (i.e. feasible and pyramidal also when the top trophic level has been deleted) in 1000 model realizations for every chain length, where the trophic interaction strengths and the consumer mortality rates have been drawn at random from defined intervals (a_{ij}=[100 0], a_{ji}=[0 1], log|b_i|=[-2.5-0.5×(i-2) -1.5-0.5×(i-2)]). Values for remaining parameters: a_{11}=1, b_1=100. (B) Same as (A) except that it shows the fraction of species-deletion pyramidal chains relative to the number of pyramidal chains (instead of the number of feasible chains as in (A)).

FIGURE S10

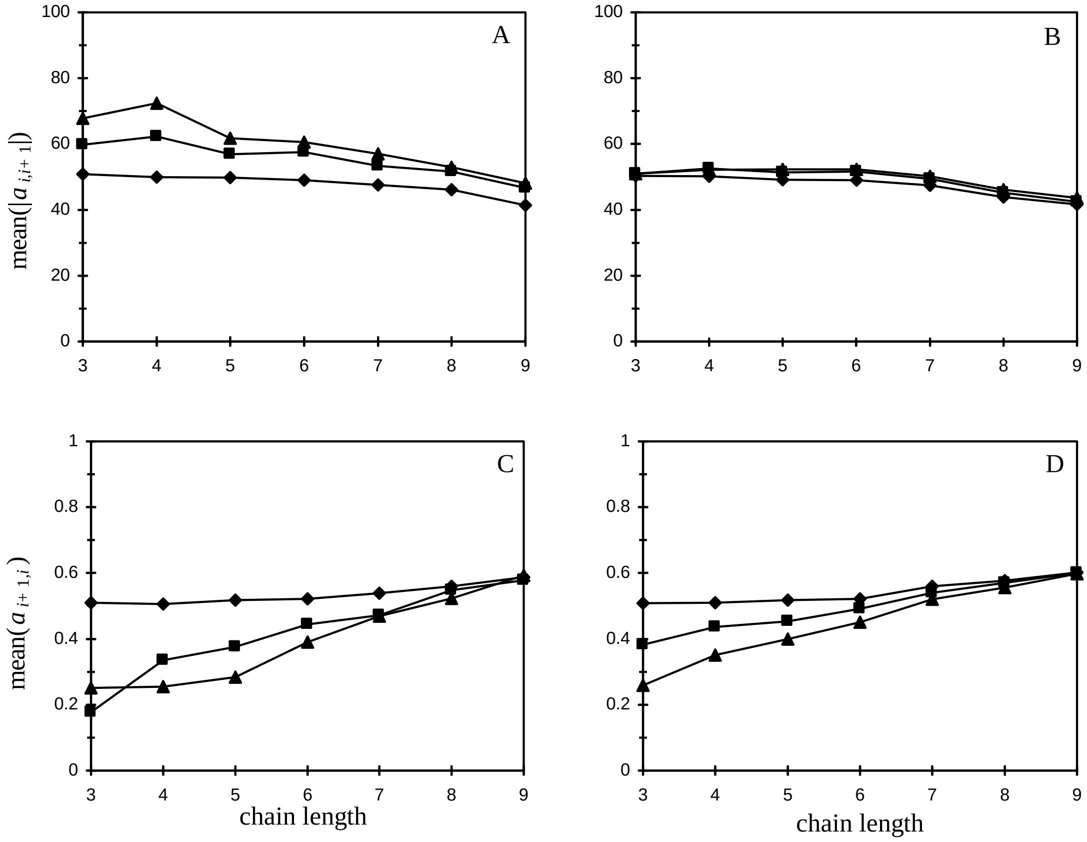

Fig. S10 The mean consumer and resource interaction strengths for the Lotka-Volterra food chains with 3 to 9 trophic levels, that are feasible (◆), pyramidal (■) and species-deletion pyramidal (▲) in 1000 realizations for every chain length, where the trophic interaction strengths and the consumer mortality rates have been drawn at random from defined intervals (a_{ij}=[100 0], a_{ji}=[0 1], log|b_i|=[-2.5-0.5×(i-2) -1.5-0.5×(i-2)]). For correlations refer to Table S2. Values for remaining parameters: (A)-(B): a_{11}=1, b_1=100, a_{ii}=0 (i≥2), (C)-(D): a_{11}=1, b_1=100, a_{ii}=0.5 (i≥2).

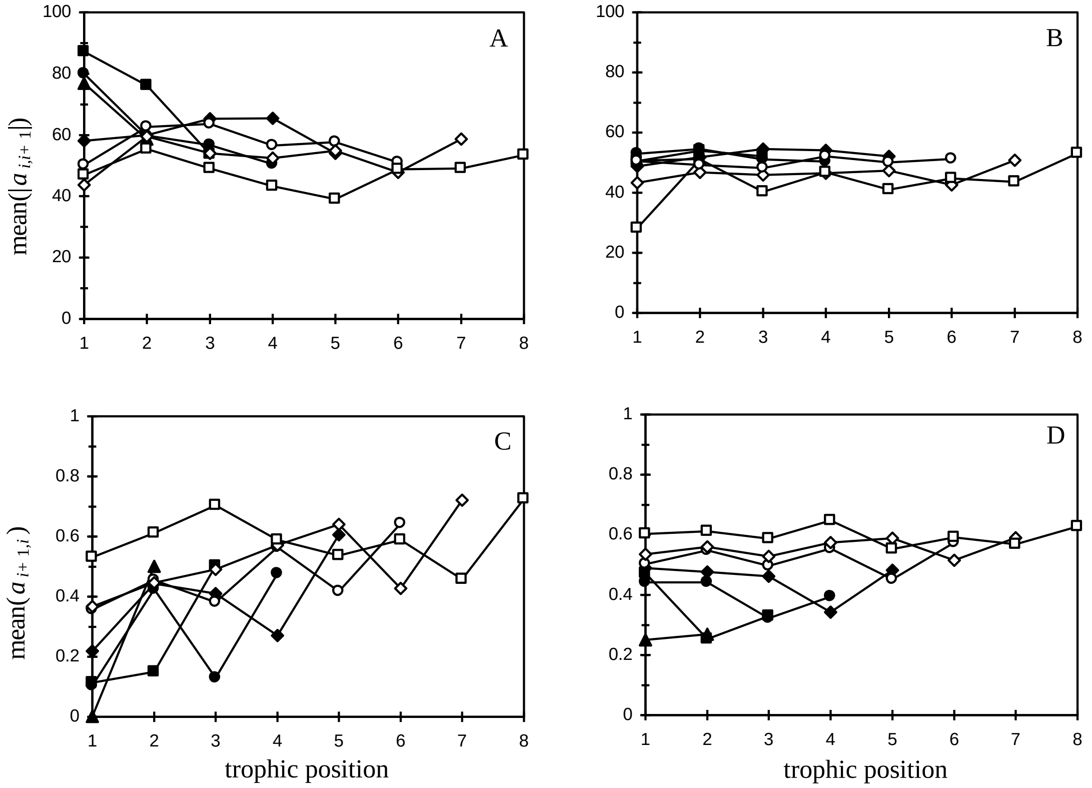

Fig. S11 The mean consumer and resource interaction strengths at different trophic positions in a food chain, for the Lotka-Volterra food chains with 3 to 9 trophic levels, that are species-deletion pyramidal in 1000 realizations for every chain length. For trophic position *n* mean($a_{n,n+1}$) and mean($a_{n+1,n}$) is shown for chains of length 3 to *n* (▲: 3, ■: 4, ●: 5, ◆: 6, ○: 7, ◇: 8, □: 9). The trophic interaction strengths and the consumer mortality rates have been drawn at random from defined intervals (a_{ij}=[100 0], a_{ji}=[0 1], log|b_i|=[-2.5-0.5×(i-2) - 1.5-0.5×(i-2)]). For correlations refer to Table S3. Values for remaining parameters: (A) & (C): a_{11}=1, b_1=100, a_{ii}=0 (i≥2), (B) & (D): a_{11}=1, b_1=100, a_{ii}=0.5 (i≥2).

FIGURE S12

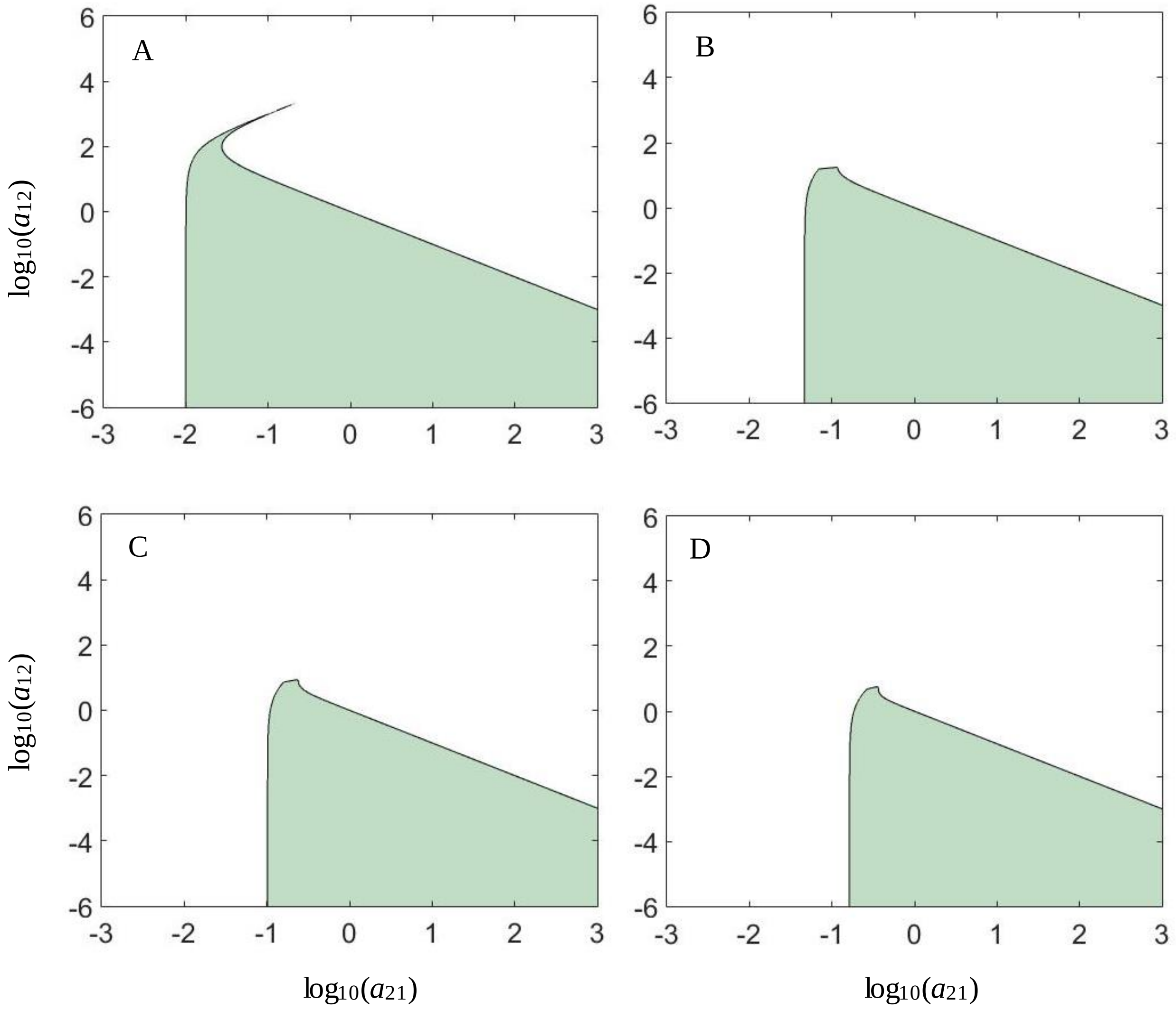

Fig. S12 Top-down vs. bottom-up control in Lotka-Volterra food chains with different number of trophic levels, with density-dependent consumer mortality Filled areas show the combinations of resource (a_{21}) and consumer (a_{12}) interaction strengths where the index in Eq. 2 is greater than unity, in Lotka-Volterra food chains with three (*A*), four (*B*), five (*C*) and six (*D*) trophic levels, and thus where the effect of self-limitation on every consumer trophic levels is stronger that that exerted by predation from the trophic level above. Parameter settings: $a_{11} = 1$, $a_{ii} = 1$ ($i \geq 2$), $b_1 = 1$, $b_i = 0.0001$ ($i \geq 2$), $a_{i,i+1} = a_{12}$ and $a_{i+1,i} = a_{21}$.

FIGURE S13

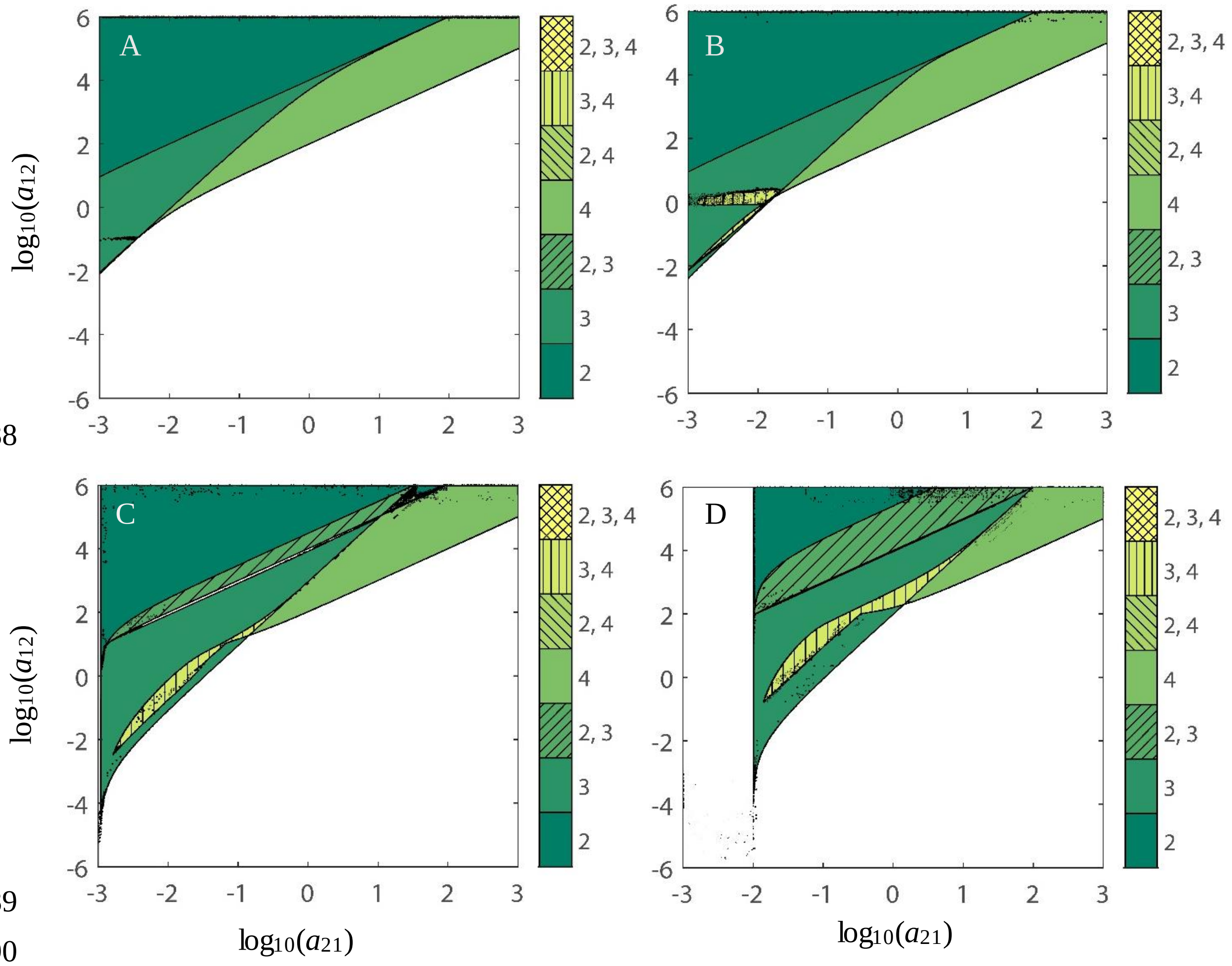

Fig. S13 The presence of Eltonian pyramids in Lotka-Volterra (LV) food chains with different number of trophic levels, where consumers have a type 2 functional response, assuming constant mortality rates and no density dependent consumer mortality. Subplots show, using different colors and hatching, the regions in parameter space (i.e. combinations of prey, a_{21}, and predator, a_{12}, interaction strengths) where LV food chains of different lengths are pyramidal. For some combinations of a_{21} and a_{12} only one food chain length will produce Eltonian pyramids (= unhatched sectors, e.g. two trophic level food chains within dark green sector), while for other combinations of a_{21} and a_{12} more than one food chain length will produce Eltonian pyramids (= hatched sectors, e.g. both two and four, but not three trophic level food chains within light green, diagonally hatched sector). See colorbar for which lengths of a food chain that are pyramidal within each sector. The functional response describes the consumption rate, $F(N_i)$, of the consumer (N_j) as a function of resource density (N_i) and is here modelled as: $F(N_i) = \dfrac{aN_i}{1 + c_j N_i}$ with (*A*) $c_j = 0.1$ ($i \geq 2$), (*B*) $c_j = 1$ ($i \geq 2$), (*C*) $c_j = 10$ ($i \geq 2$), (*D*) $c_j = 100$ ($i \geq 2$). Other parameters: $a_{11} = 1$, $a_{ii} = 0$ ($i \geq 2$), $b_1 = 1$, $b_i = 0.0001$ ($i \geq 2$), $a_{i,i+1} = a_{12}$ and $a_{i+1,i} = a_{21}$. The food chain model with saturating functional responses could not be solved analytically. Instead, equilibrium abundances had to be searched for

numerically here and black spots in subplots represent areas where the numerical solver (fsolve in Matlab) could not find an explicit solution (but where the solution for mathematical reasons most likely was similar to that in the surronding area).

FIGURE S14

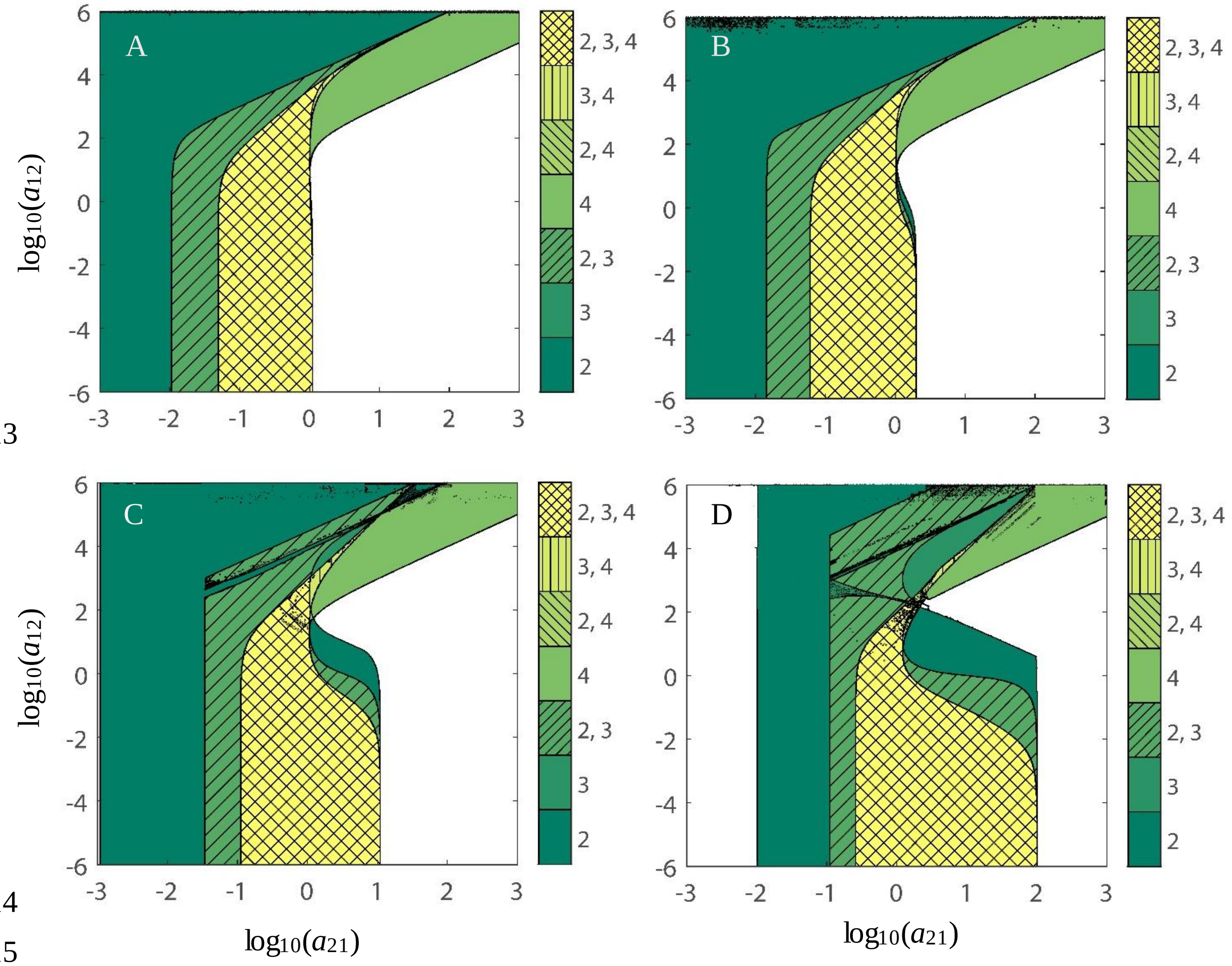

Fig. S14 Same as Fig. S13 except for inclusion of density dependent consumer mortality (i.e. $a_{ii} = 1$, $i \geq 2$).

TABLE S1

THE EFFECT OF DENSITY-DEPENDENT CONSUMER MORTALITY ON THE PYRAMID OF NUMBERS IN LOTKA-VOLTERRA FOOD CHAINS

a_{ii}	Chain Length	Number Feasible	Number Pyramidal	Number Species-Deletion Feasible	Number Species-Deletion Pyramidal
0	3	998	17	17	3
0.5	3	999	510	510	252
0	4	994	61	61	3
0.5	4	988	534	534	266
0	5	931	60	60	9
0.5	5	913	516	516	302
0	6	873	222	222	16
0.5	6	812	588	588	369
0	7	613	116	116	36
0.5	7	532	385	385	274
0	8	422	168	168	30
0.5	8	299	244	244	184
0	9	190	46	46	18
0.5	9	110	92	92	78

NOTE.— The table shows the number of feasible, pyramidal, species-deletion feasible and species-deletion pyramidal model chains (see Note S5 for definition) out of 1000 replicates, where the mortality rates and trophic interaction strengths of Eq. (1) where drawn at random from specified intervals. The model is a Lotka-Volterra food chain model with (a_{ii}=0.5) and without (a_{ii}=0) density-dependent consumer mortality. Parameter settings used: b_1=100, a_{11}=1, $\log|b_i|$ ($i \geq 2$) was drawn at random from the interval $\log|b_i|=[(i-2)*k+\log|b_2|-0.5\ (i-2)*k+\log|b_2|+0.5]$, where k=-0.5, $\log|b_2|$=2 and i is the trophic position in the chain. Consumer and resource interaction strengths were drawn at random from the intervals [100 0] and [0 1] respectively.

TABLE S2

Correlation Between Chain Length and Mean Consumer and Resource Interaction Strengths in Lotka-Volterra Food Chains

	a_{ii}=0			a_{ii}=0.5		
	Feasible Chains	Pyramidal Chains	Species-Deletion Pyramidal Chains	Feasible Chains	Pyramidal Chains	Species-Deletion Pyramidal Chains
mean($\lvert a_{i,i+1} \rvert$)	-0.9079***	-0.9355**	-0.9499**	-0.9306**	-0.8596*	-0.8122*
mean($a_{i+1,i}$)	0.9346**	0.9707***	0.9788***	0.9493**	0.9958***	0.9923***

NOTE.—Presented in the table are the coefficients of correlation for the relationship between chain length and mean consumer ($\lvert a_{i,i+1} \rvert$) and resource ($a_{i+1,i}$) trophic interaction strengths shown in Fig. S10, for model food chains of Lotka-Volterra type with (a_{ii}=0.5, $i \geq 2$) and without (a_{ii}=0, $i \geq 2$) density-dependent consumer mortality (with trophic interaction strengths and mortality rates drawn at random from specified intervals; see text and legend to Fig. S10). Feasible chains are the fraction out of 1000 randomizations that have a feasible equilibrium for every species, pyramidal chains are the fraction with a pyramidal abundance pattern and species-deletion pyramidal are those pyramidal chains that can lose the top species and still have a pyramidal abundance pattern. For parameter settings refer to legend to Fig. S10.

*=p<0.05, **=p<0.01, ***=p<0.001.

TABLE S3

CORRELATION BETWEEN TROPHIC POSITION AND MEAN CONSUMER AND RESOURCE INTERACTION STRENGTHS IN LOTKA-VOLTERRA FOOD CHAINS

		Chain Length					
		4	5	6	7	8	9
a_{ii}=0	mean($\|a_{i,i+1}\|$)	-0.9810†	-0.9261‡	-0.0871¶	-0.1655¶	0.3008¶	0.0411¶
	mean($a_{i+1,i}$)	0.9042§	0.5484§	0.6211§	0.7180†	0.7166‡	0.0643¶
a_{ii} =0.5	mean($\|a_{i,i+1}\|$)	0.4276¶	-0.7701§	0.6210§	0.3727¶	0.4435§	0.5490†
	mean($a_{i+1,i}$)	-0.6402¶	-0.6032§	-0.3820¶	0.1575¶	0.3447§	0.1216¶

NOTE.—The table shows the coefficient of correlation for the relationship between trophic position and mean consumer ($|a_{i,i+1}|$) and resource ($a_{i+1,i}$) trophic interaction strengths shown in Fig. S11, for model food chains of Lotka-Volterra type, with (a_{ii}=0.5, i≥2) and without (a_{ii}=0, i≥2) density-dependent consumer mortality, that are species-deletion pyramidal (with trophic interaction strengths and mortality rates drawn at random from specified intervals, see text and legend to Fig. S11). Species-deletion pyramidal chains are those pyramidal chains (out of 1000 randomizations) that can lose the top species and still have a pyramidal abundance pattern.

¶=p>0.5, §=p<0.5, †=p<0.2, ‡=p<0.1.
